# Cord Blood Exosomal miRNAs from Small-for-Gestational-Age Newborns: Association with Measures of Postnatal *Catch-Up* Growth and Insulin Resistance

**DOI:** 10.3390/ijms26146770

**Published:** 2025-07-15

**Authors:** Marta Díaz, Tania Quesada-López, Francesc Villarroya, Abel López-Bermejo, Francis de Zegher, Lourdes Ibáñez, Paula Casano-Sancho

**Affiliations:** 1 Endocrinology Department, Institut de Recerca Sant Joan de Déu, University of Barcelona, 08950 Barcelona, Spain; lourdes.ibanez@sjd.es; 2 Centro de Investigación Biomédica en Red de Diabetes y Enfermedades Metabólicas Asociadas (CIBERDEM), Instituto de Salud Carlos III, 28029 Madrid, Spain; 3 Department of Biomedicine, Institut de Recerca Hospital de la Santa Creu i Sant Pau, 08041 Barcelona, Spain; qtania@gmail.com; 4 Network Biomedical Research Center of Physiopathology of Obesity and Nutrition (CIBEROBN), Health Institute Carlos III, 28029 Madrid, Spain; fvgombau@gmail.com; 5 Biochemistry and Molecular Biomedicine Department, Institute of Biomedicine, University of Barcelona, 08950 Barcelona, Spain; 6 Institut de Recerca Sant Joan de Déu, 08950 Esplugues, Spain; 7 Pediatric Endocrinology Research Group, Girona Institute for Biomedical Research (IDIBGI), Faculty of Medicine, University of Girona and Dr. Josep Trueta Hospital, 17190 Girona, Spain; alopezbermejo@idibgi.org; 8 Leuven Research & Development, University of Leuven, 3000 Leuven, Belgium; francis.dezegher@kuleuven.be

**Keywords:** miRNAs, exosomes, SGA, insulin resistance, body composition, DXA

## Abstract

Small-for-gestational-age (SGA) infants who experience a marked postnatal *catch-up*, mainly in weight, are at risk for developing metabolic disorders; however, the underlying mechanisms are imprecise. Exosomes and their cargo (including miRNAs) mediate intercellular communication and may contribute to altered crosstalk among tissues. We assessed the miRNA profile in cord blood-derived exosomes from 10 appropriate-for-gestational-age (AGA) and 10 SGA infants by small RNA sequencing; differentially expressed miRNAs with a fold change ≥2.4 were validated by RT-qPCR in 40 AGA and 35 SGA infants and correlated with anthropometric, body composition (DXA) and endocrine–metabolic parameters at 4 and 12 mo. miR-1-3p, miR-133a-3p and miR-206 were down-regulated, whereas miR-372-3p, miR-519d-3p and miR-1299 were up-regulated in SGA infants. The target genes of these miRNAs related to insulin, RAP1, TGF beta and neurotrophin signaling. Receiver operating characteristic analysis disclosed that these miRNAs predicted with accuracy the 0–12 mo changes in body mass index and in total and abdominal fat and lean mass. In conclusion, the exosomal miRNA profile at birth differs between AGA and SGA infants and associates with measures of *catch-up* growth, insulin resistance and body composition through late infancy. Further follow-up of this population will disclose whether these associations persist into childhood, puberty and adolescence.

## 1. Introduction

Exosomes are small extracellular vesicles secreted by most tissues and present in all biofluids [1]. Exosomes carry lipids, proteins, DNA and a variety of RNA species, including mRNA and non-coding RNAs (miRNAs, lncRNAs and circRNAs), that act as mediators of cell-to-cell communication in both a paracrine- and endocrine-like manner [2]. miRNAs have a critical role in the post-transcriptional regulation of gene expression [3] and are the most studied RNA cargo regarding function and impact on intercellular communication. Exosomal functional transfer of miRNAs has been demonstrated in in vitro and in vivo models for different disorders and physiological states, inducing a broad range of downstream effects [4,5,6]. Notably, exosomal miRNAs exhibit different expression patterns in physiological and pathological states and have emerged as potential diagnostic and therapeutic tools for several human diseases [1,7,8]. A plethora of studies support the biological effects of exosomes in obesity, type 2 diabetes (T2D), insulin resistance (IR) and metabolic dysfunction-associated steatotic liver disease (MASLD) [9,10,11,12,13]. Moreover, placental-derived exosomes play a pivotal role in embryo/feto-maternal communication [14]. Indeed, an altered exosome number and exosomal miRNA profile have been reported in gestational diabetes, preeclampsia and intrauterine growth restriction [15]. In this context, miRNAs delivered via exosomes from the placenta and/or maternal tissues might permanently alter the expression levels of genes in target cells affecting fetal organ development, including in the pancreas, liver and adipose tissue, and thus in key metabolic pathways that shape lifelong metabolic health.

Small-for-gestational-age infants (SGA; birth weight < −2 SD for gestational age) who experience a rapid and excessive postnatal *catch-up* in weight are at increased risk for developing metabolic disturbances, including IR, hepato-visceral fat depots, T2D, and cardiovascular disorders later in life [16,17,18]. However, the mechanisms underpinning these outcomes are not fully understood.

SGA infants display at birth exosomes that are larger in size [19] and have a qualitatively and quantitatively distinct proteome content than those born appropriate-for-GA (AGA) [20]. Here, we analyze the expression patterns of cord blood-derived exosomal miRNAs in AGA vs. SGA infants to disclose their relation with prenatal growth and postnatal outcomes into late infancy and to find out whether differentially expressed miRNAs could serve as early biomarkers of rapid weight gain and fat accretion over the first year of life.

## 2. Results

### 2.1. Characteristics of the Study Population

Table 1 depicts anthropometric, endocrine–metabolic and body composition parameters according to birth weight. As previously reported, at birth SGA infants were lighter and shorter and had less total and abdominal fat mass, less lean mass and lower circulating levels of insulin-like growth factor-1 (IGF-1) and high-molecular-weight adiponectin (HMW-adip) as compared to AGA infants [19,20,21]. At age 4 months, SGA infants remained smaller but experienced a *catch-up* in fat mass accompanied by increasing levels of IGF-1 and HMW-adip. At age 12 months, SGA infants completed their *catch-up* in weight and length and normalized HMW-adip circulating levels, but still had less lean mass and higher circulating IGF-1 concentrations.

### 2.2. Exosomal miRNA Expression Profile

**Figure 1:**
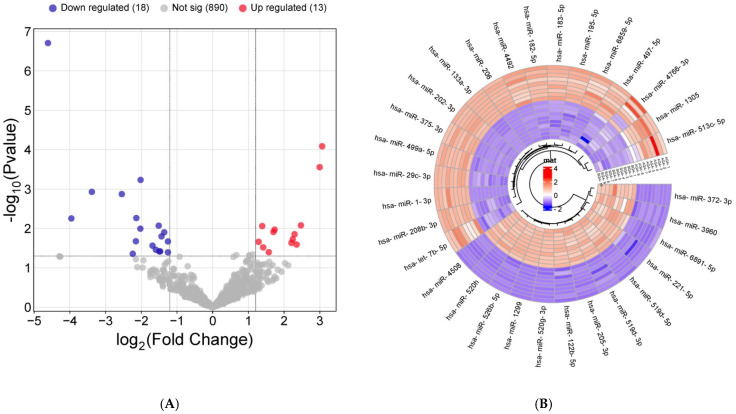
miRNAs differentially expressed in cord blood-derived exosomes from SGA compared with AGA infants by RNA sequencing. (**A**) Volcano plot showing statistical significance (−log_10_ *p*-value) versus fold change (log_2_ fold change) of RNA-seq data from AGA, (n = 9) vs. SGA (n = 10) infants. Up-regulated miRNAs (log_2_ fold change value ≥ 1.3 and *p*-value < 0.05 by unpaired *t*-test) are shown in red, and down-regulated miRNAs (fold change value ≤ −2.4 or ≥2.4 and *p*-value < 0.05 by unpaired *t*-test) are shown in blue. Non-differentially expressed miRNAs between AGA and SGA subgroups are shown in grey (**B**) A hierarchically clustered heatmap showing the expression patterns of the 31 differentially expressed miRNAs. Red and blue represent up- and down-regulated expression, respectively, in the AGA and SGA subgroups. Color density indicates expression levels.

Small RNA sequencing was successfully performed in n = 9 AGA and n = 10 SGA cord blood-derived exosomal samples and identified a total of 1241 miRNAs. Of these, 921 miRNAs were shared by the AGA and SGA subgroups (Figure 1A); 125 miRNAs were exclusively detected within the AGA subgroup (Table S1); and 195 miRNAs were only found in the SGA subgroup (Table S2). Thirty-one miRNAs (out of 921) were dysregulated in SGA infants (n = 18 down-regulated and n = 13 up-regulated) (Figure 1A,B).

**Figure 2:**
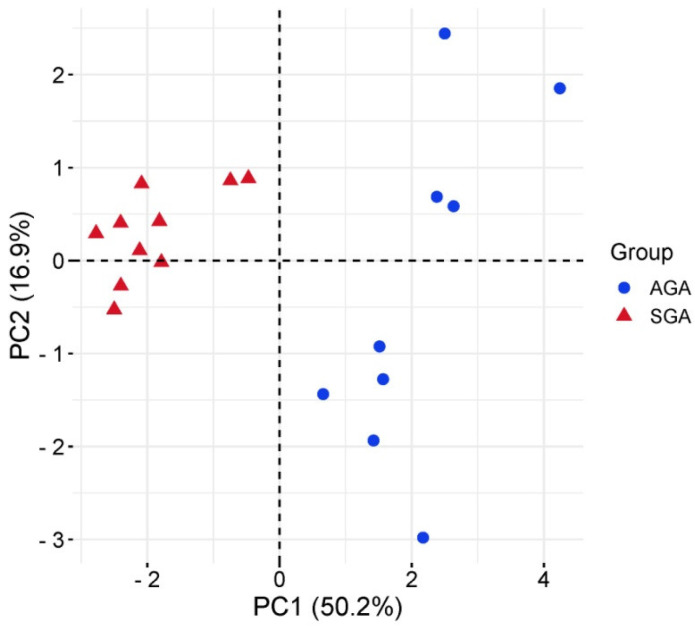
Principal component analysis (PCA) of the miRNA profile in cord blood-derived exosomes from AGA (n = 9, blue dots) and SGA (n = 10, red triangles) infants.

Principal component analysis (PCA) showed a clear segregation between subgroups, with PC1 and PC2 explaining 67% of the variance (Figure 2).

**Figure 3:**
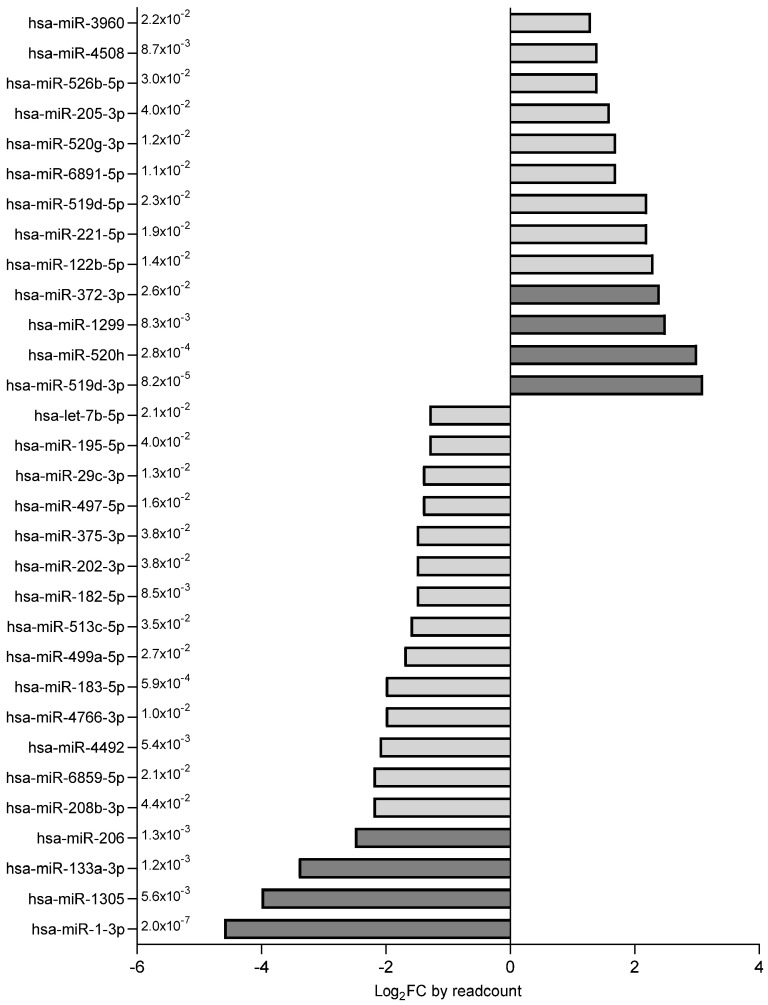
Differentially expressed miRNAs (n = 31) were ranked according to their fold change. miRNAs depicted in dark grey (FC ≤ −2.4 or ≥2.4, *p* < 0.05) were selected for validation in the entire study population of AGA (n = 40) and SGA (n = 35) infants.

**Figure 4:**
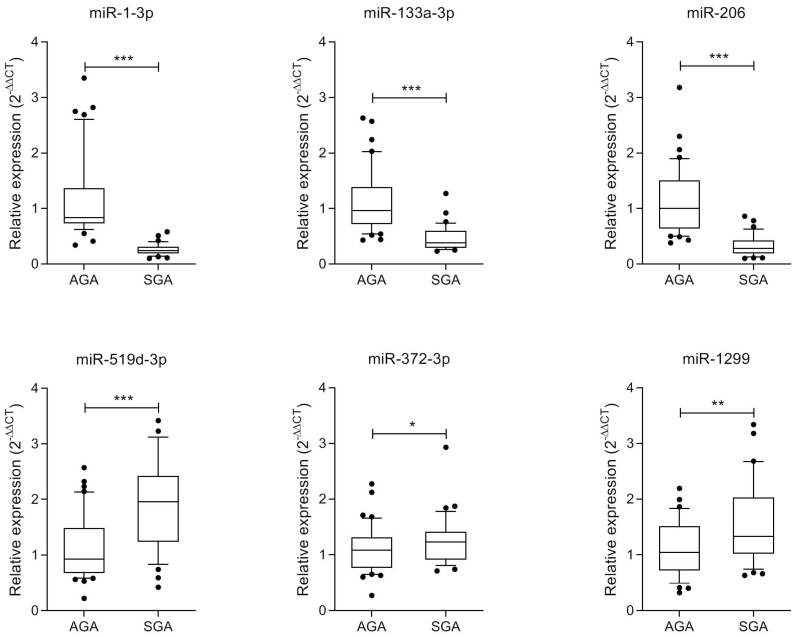
Boxplots (median and interquartile ranges) of miRNAs confirmed as differentially expressed in cord blood-derived exosomes from AGA (n = 40) and SGA (n = 35) infants. Whiskers represent centiles 10 and 90. * *p* = 0.029; ** *p* = 0.007; *** *p* < 0.0001.

The miRNAs with the higher fold change (n = 8, FC ≤ −2.4 or ≥2.4) (Figure 3) were further verified in the whole study population; 6 out of these 8 miRNAs were confirmed as differentially expressed (Figure 4). Among them, miR-1-3p, miR-133a-3p and miR-206 were down-regulated, whereas miR-519d-3p, miR-372-3p and miR-1299 were up-regulated in the SGA subgroup (Figure 4). The remaining two miRNAs, miR-1305 and miR-520h, were comparable between groups.

### 2.3. Target Genes of Differentially Expressed miRNAs

To predict potential targets for the validated miRNAs, we used the miRSystem database. The software predicted 725 target genes for miR-1-3p, 392 for miR-133a-3p, 699 for miR-206, 1000 for miR-519d-3p and 814 for miR-372-3p. No target genes were predicted for miR-1299, so we checked them with miRDB, another bioinformatics tool, which disclosed 1119 potential targets.

### 2.4. Pathway Analysis of Differentially Expressed miRNAs

**Figure 5:**
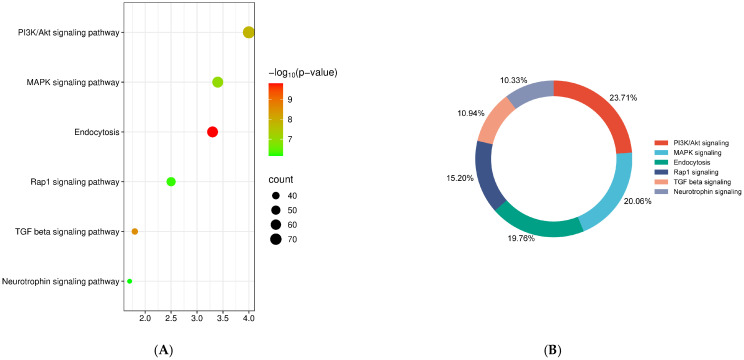
Pathway enrichment analysis. (**A**) KEGG pathway analysis with the targets of all differentially expressed exosomal miRNAs. (**B**) Percentage of target genes involved in the top most enriched pathways.

The database for annotation, visualization and integrated discovery (DAVID) was used to perform pathway enrichment analysis of predicted targets (Table 2). KEGG pathway analysis, including the target genes of all differentially expressed miRNAs, revealed the top six most enriched pathways, namely, phosphatidylinositol 3-kinase/protein kinase B (PI3K-Akt) signaling, mitogen-activated protein kinase (MAPK) signaling, endocytosis, transforming growth factor (TGF) beta signaling, Ras-associated protein 1 (RAP1) signaling and neurotrophin signaling (Figure 5A). Insulin signaling accounted for 44% of total target genes, followed by endocytosis (20%), TGF beta signaling (11%), RAP1 signaling (15%) and neurotrophin signaling (10%) (Figure 5B).

### 2.5. miRNA–Target Gene Network

**Figure 6:**
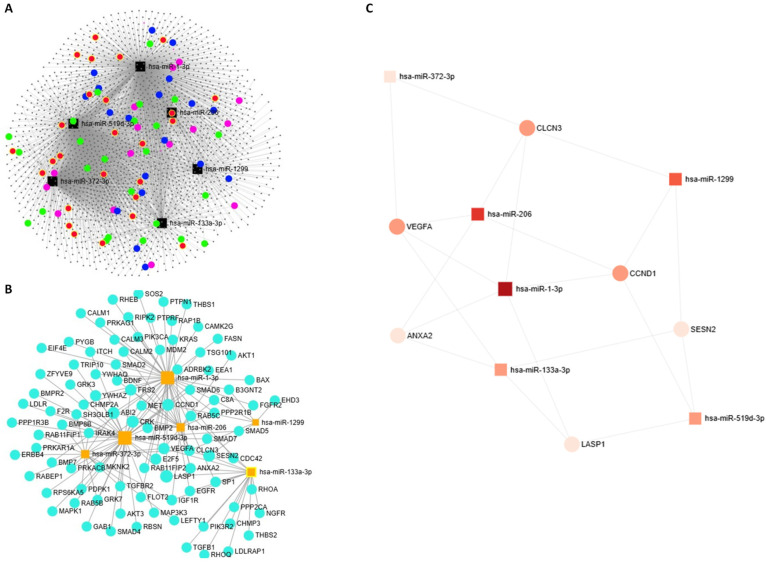
miRNA–mRNA regulatory network between differentially expressed exosomal miRNAs in SGA vs. AGA infants. (**A**) miRNA–target gene topology network for differentially expressed exosomal miRNAs. The grey nodes represent genes, and the black nodes represent miRNAs. Green, red, blue and pink nodes are genes related to endocytosis, insulin signaling, TGF beta signaling and neurotrophin signaling, respectively. (**B**) Network of the target genes (blue dots) of the selected exosomal miRNAs (orange squares) related to endocytosis, insulin signaling, TGF beta signaling and neurotrophin signaling. (**C**) Network of the hub target genes.

The differentially expressed miRNAs were analyzed using miRNet 2.0 to construct a miRNA–target gene network. All miRNAs showed a degree ≥3 (number of connections of each miRNA with other miRNAs). This network revealed that these miRNAs were linked to 2140 genes which were visually analyzed as a topology network (Figure 6A). The miRNA–mRNA network of the hub genes related to endocytosis, insulin signaling, TGF beta signaling and neurotrophin signaling is displayed in Figure 6B. The global network with the hub target genes (genes with the most important roles, as judged by the multiple interactions with other genes) is depicted in Figure 6C.

### 2.6. Receiver Operating Characteristics (ROC) Analysis

The ROC curve analysis disclosed that the six differentially expressed miRNAs showed the potential to differentiate the subgroups according to birth weight. Namely, mir-1-3p, mir-133a-3p and mir-206 [area under the curve (AUC) 0.99 (0.98–1.00), 0.92 (0.97–0.98), and 0.95 (0.91–0.99), respectively, all *p* < 0.0001], displayed an outstanding discrimination power (Figure S1A), while mir-519d-3p, mir-372-3p and mir-1299 [AUC 0.78 (0.67–0.88), *p* < 0.0001; 0.65 (0.52–0.77), *p* = 0.029; and 0.68 (0.56–0.80), *p* = 0.007, respectively] showed an acceptable discrimination capacity (Figure S1A).

In addition, all miRNAs exhibited a remarkable capacity to predict the 0–1 year changes in body mass index (BMI) [AUC 0.97 (0.91–1.0), for mir-1-3p, mir-133a-3p and mir-206, all *p* < 0.0001; 0.92 (0.88–0.97), *p* < 0.0001 for mir-519d-3p; 0.96 (0.92–0.99), *p* < 0.0001 for miR-372-3p; and 0.95 (0.91–0.99), *p* < 0.0001 for mir-1299] (Figure S1B); in fat mass [AUC 0.96 (0.93–1.0), *p* < 0.0001 for mir-1-3p; 0.98 (0.96–1.0) for mir-133a-3p, mir-206 and mir-372-3p, all *p* < 0.0001; 0.86 (0.81–0.93), *p* < 0.0001 for mir-519d-3p; and 0.93 (0.89–0.97), *p* < 0.0001 for mir-1299] (Figure S1C); abdominal fat [AUC 0.91 (0.87–0.96), *p* < 0.0001 for mir-1-3p; 0.99 (0.98–1.0), *p* < 0.0001 for mir-133a-3p; 0.93 (0.89–0.97), *p* < 0.0001 for mir-206; and 0.99 (0.99–1.0) for mir-519d-3p, miR-372-3p and mir-1299, all *p* < 0.0001] (Figure S1D); and in lean mass as well [AUC 0.97 (0.94–1.0) for mir-1-3p, mir-133a-3p and mir-206, all *p* < 0.0001; 0.92 (0.87–0.97), *p* < 0.0001 for mir-519d-3p; 0.96 (0.92–1.0), *p* < 0.0001 for miR-372-3p; and 0.95 (0.91–0.99), *p* < 0.0001 for mir-1299] (Figure S1E). Moreover, all miRNAs displayed a good capacity for predicting the 0–1 year changes in HOMA-IR [AUC 0.75 (0.66–0.85), *p* < 0.0001 for mir-1-3p; 0.77 (0.68–0.87), *p* < 0.0001 for mir-133a-3p; 0.76 (0.67–0.85), *p* < 0.0001 for mir-206; 0.85 (0.78–0.92), *p* < 0.0001 for mir-519d-3p; 0.82 (0.74–0.91), *p* < 0.0001 for mir-372-3p; and 0.84 (0.76–0.92), *p* < 0.0001 for mir-1299] (Figure S1F).

### 2.7. Correlation Analysis

At birth, expression levels of down-regulated mir-1-3p, mir-133a-3p and mir-206 were directly associated with gestational age, weight, length and BMI (and derived Z-scores), with IGF-1 and circulating HMW-adip levels, and with fat mass, abdominal fat and lean mass (Table 3). Down-regulated miRNAs showed, in addition, a negative correlation with the changes at 0–4 mo and 0–12 mo in weight and BMI Z-score and in circulating IGF-1 and HMW-adip levels (Table 3).

At birth, up-regulated miRNAs were negatively associated with gestational age, weight and weight Z-score, and with BMI Z-score (Table 4). The circulating levels of IGF-1 and HOMA-IR inversely correlated with mir-372-3p expression levels, whereas mir-519d-3p and mir-1299 were negatively associated with fat and lean mass (Table 4). mir-519d-3p, mir-372-3p and mir-1299 expression levels positively correlated with the 0–4 mo and 0–12 mo changes in weight Z-score, BMI and BMI Z-score, circulating HOMA-IR and IGF-1 levels, and total and abdominal fat (Table 4).

## 3. Discussion

To our knowledge, this is the first study assessing the miRNA profile in cord blood-derived exosomes from AGA vs. SGA infants and reporting the associations with anthropometric measures, endocrine–metabolic markers and body composition variables over the first year of life. Our results strengthen the notion that microRNAs may become promising biomarkers for the early prediction of adverse metabolic outcomes in this population.

We discovered a decreased expression of miR-1-3p, miR-133a-3p and miR-206 and an overexpression of miR-519d-3p, miR-372-3p and miR-1299 in cord blood-derived exosomes from SGA infants. The target genes of these miRNAs were mainly involved in insulin, RAP1, TGF beta, and neurotrophin signaling, and in endocytosis. The miRNA–gene interaction network further identified six hub genes (*CLCN3*, *VEGFA*, *ANXA2*, *LASP1*, *SESN2* and *CCND1*) as key regulators of those processes.

A few studies have disclosed the associations between circulating miRNAs at birth and prenatal [22,23] or in postnatal growth and metabolic outcomes in early childhood [24,25], with apparently diverging results. However, those studies were performed on a different type of sample or used as a definition for SGA a criterion different from a birth weight of <−2 SD for gestational age and sex, so overlap between groups would be expected. Moreover, all of them analyzed non-exosomal miRNAs using different methodologies [24,25]. The source of miRNAs (cellular vs. vesicle or cell-free miRNAs) being the cause of variation in results has been explored by Reithmair et al. [26]. It is worth noting that the exosomal source of miRNAs has been shown to be the gold standard method for biomarker studies owing to its benefits in terms of quantity, quality and stability [27], also allowing the detection of low-abundance molecules of interest [28]. miR-1-3p, miR-133a-3p and miR-206, the so-called “myo-miRs”, are a specific group of muscle-enriched miRNAs that play key roles in skeletal and cardiac muscle development, function and regeneration and in myogenesis [29]. At birth, SGA infants were less adipose and had less lean mass than AGA infants; in late infancy, the relative deficit in lean mass persisted, while the amount of body adiposity was similar to that of AGA infants. Skeletal muscle represents about 40–45% of body weight and has a key role in glucose and oxidative metabolism, so that a deficit in muscle mass may significantly impact whole-body metabolism [30] and trigger the development of insulin resistance (IR) and associated comorbidities, contributing to the increased metabolic risk in SGA subjects [31].

Myo-miRs, and miR-519d-3p, miR-372-3p and miR-1299, also play a role in the regulation of energy metabolism and insulin signaling in skeletal muscle, white adipose tissue (WAT) and hepatocytes. Decreased expression of miR-1-3p and miR-133a-3p has also been found in skeletal muscle of IR mice through altered IGF-1-mediated signaling [32]. Of note, IGF-1 and its receptor are targets of miR-1-3p and miR-133a-3p, and a feedback loop between myo-miR expression and IGF-1 signal transduction has been revealed [32]. Thus, the negative associations between myo-miR expression and IGF-1 levels and BMI/BMI Z-score increase at 0–12 months suggest that myo-miR-mediated regulation of IGF-1 receptor signaling could be among the mediators of postnatal *catch-up* in weight. The higher IGF-1 concentrations displayed by SGA infants at 4 and 12 months, and the negative correlations between myo-miRs and IGF-1 levels, strengthen this hypothesis.

Differentially expressed miRNAs were also associated with 0–12-month changes in HOMA-IR. The progression from insulin sensitivity at birth to an IR state has been characterized in SGA children from the age of three to seven years [20,33]. These findings fit well with previous studies reporting a down-regulation of circulating miR-1-3p and miR-133a-3p and an up-regulation of miR-372-3p levels in T2D [34,35]; moreover, miR-133a-3p targets LIM and SH3 protein1 (LASP1), a cytoskeletal protein with a scaffolding function that interacts with AKT promoting the PI3K/AKT signaling pathway [36]. In addition, miR-372-3p directly targets FGF-16, which confers cytoprotection against high glucose-induced endothelial disorders [35], and vascular endothelial growth factor A (VEGFA), which is needed for normal glucose-stimulated insulin secretion [37,38]; on the other hand, miR-206 targets Annexin A2 (ANXA2) a calcium-dependent phospholipid-binding protein with a key role in MASLD [39]. Furthermore, Sestrin2 (SESN2), an upstream activator of AMP-activated protein kinase (AMPK) and repressor of rapamycin complex 1 (mTORC1), has been show to control both glucose and lipid metabolism [40]. In addition, recent studies have disclosed the association between miR-1299 expression levels and prediabetes, T2D, and gestational diabetes [41,42]. Furthermore, miR-1299 targets cyclin D1 (CCND1) [43] and the CCND1-CDK4 complex—which is activated by insulin—has a key role in the regulation of glucose metabolism [44].

Dysregulation of myo-miRs may play a role in the associations between birth size and cardiometabolic risk [45]. For example, Clcn3 (chloride channel/antiporter) is directly targeted by miR-1-3p, and heart-specific inducible *Clcn3* knock-out mice develop myocardial hypertrophy and reduced cardiac function [46]. miR-519d-3p is induced during adipogenesis and represses peroxisome proliferator-activated receptor alpha (PPARα) translation (with a key role in fatty acid homeostasis), suggesting that miR-519d may be involved in adipocyte hypertrophy in human obesity [47]. It is tempting to speculate that the identified miRNAs could also account for the liver fat depots already present in SGA children at age 7 yr [20]. In this regard, miR-206 inhibits hepatosteatosis and hyperglycemia in MASLD by regulating phosphatase non-receptor type 1 (PTPN1), as well as its downstream sterol regulatory element-binding protein 1c (SREBP1C) signaling pathway [48]; in addition, miR-372-3p decreases AE binding protein 1 expression levels, a central regulator driving fibrosis in MASLD [49]. Finally, steatotic hepatocytes secrete miR-1-3p containing extracellular vesicles that promote endothelial inflammation and atherosclerosis [50].

Enrichment analysis revealed that the target genes of the differentially expressed miRNAs were, overall, involved in the regulation of insulin signaling and energy balance. Indeed, impairment of PI3K/AKT and MAPK signaling may affect glucose and lipid homeostasis [51,52], whereas RAP1 signaling participates in the mechanistic pathway linking overnutrition to obesity and metabolic disorders [53].

Moreover, several neurotrophins play a key role in the regulation of food intake and energy expenditure [54]. Furthermore, smad3 deletion (a downstream protein of the TGF beta pathway) protects mice from diet-induced obesity and diabetes [55] and reverts IR in the liver, adipose tissue and skeletal muscle in db/db mice [56].

In addition, both RAP1 and TGF beta are central regulators of trophoblast invasion and placental vascularization, modulating placental development and function through the activation of PI3K/Akt and MAPK signaling. Dysregulation of these pathways would impact in nutrient transport and might contribute to fetal growth restriction [57,58] and to the development of insulin resistance later in life. Along these lines, systemic blockade of RAP1 decreases blood glucose and improves glucose tolerance in high-fat diet-induced obese mice [59], whereas systemic inhibition of TGF beta signaling increases insulin sensitivity and energy expenditure [55].

Endocytosis plays a crucial role in the regulation of intracellular signaling cascades and in exosome uptake by recipient cells [60].

In conclusion, the exosomal miRNA profile of SGA infants at birth differs from that of AGA infants, and is associated with measures of postnatal *catch-up* growth, IR, and body adiposity into late infancy. Further follow-up of this population will disclose to what extent these associations persist into childhood, puberty and adolescence.

## 4. Materials and Methods

### 4.1. Study Population

The initial study population consisted of 75 infants [40 AGA (48% girls) and 35 SGA (51% girls)], in whom the profile of circulating exosomes at birth and at age 2 and 7 yr as well as the proteome of circulating exosomes at birth had been previously assessed [19,20], (Flowchart, Figure S2). Those infants were originally enrolled into two longitudinal prospective studies conducted at Hospital Sant Joan de Déu, Barcelona [21,61,62] (Flowchart, Figure S2). As described, the inclusion criteria were as follows: singleton; uncomplicated pregnancy at term (37–42 weeks); birth weight Z-score between −1.0 SD and +1.0 SD for AGA and <−2.0 SD for SGA [19,21]; spontaneous *catch-up* in weight and length in SGA subjects, defined as weight and length Z-score > −2.0 by age 1 yr [63]; exclusive breast- or formula feeding for at least 4 months; and written informed consent. Exclusion criteria were maternal hypertension, preeclampsia or diabetes mellitus, alcohol or drug abuse, congenital malformations, or complications at birth.

### 4.2. Assessments

Gestational age was calculated according to the last menses and confirmed by first-trimester ultrasound. Weight and length were measured at birth and at 4 and 12 months, and BMI was derived at each time point. Weight, length and BMI Z-scores were derived from country and sex-specific references, as described [64].

Blood samples were obtained at birth from the umbilical cord before placental expulsion and in the morning before the first feed of the day at 4 and 12 months. Serum samples were kept at −80 °C until analysis.

Serum glucose was measured by the glucose oxidase method. Circulating insulin and IGF-1 were measured by immuno-chemiluminescence (DPC IMMULITE 2500, Siemens, Erlangen, Germany). IR was estimated using the homeostatic model assessment (HOMA-IR) (fasting insulin (mU/L) × fasting glucose (mmol/L)/22.5). Circulating HMW-adip was measured using a specific human ELISA kit (R&D Systems, Minneapolis, MN, USA). The intra- and inter-assay coefficients of variation (CVs) were <9%.

Body composition was assessed at age 15 days and at 12 months, by dual X-ray absorptiometry (DXA) with a Lunar Prodigy coupled to Lunar software, version 3.4/3.5 (Lunar Corp., Madison, WI, USA), adapted for infants [21]; fat and lean mass CVs were <3%.

### 4.3. RNA Isolation

RNA was isolated from 100 µL of exosome fractions purified from the cord blood of 40 AGA and 35 SGA infants using the miRNeasy Serum/Plasma kit (Qiagen, Germantown, MD, USA) according to the manufacturer’s instructions, and with an elution volume of 14 µL.

### 4.4. Library Preparation and miRNA Sequencing

10 AGA and 10 SGA RNA samples were selected for miRNA profiling by RNA-sequencing (Qiagen) (Flowchart, Figure S2), ensuring representation of the entire cohort (40 AGA, 35 SGA) in terms of sex, gestational age and birth weight.

A miRNA sequencing library was prepared from 5 µL total RNA using the QIAseq miRNA Library Kit (Qiagen, Venlo, The Netherlands). A pre-adenylated DNA adapter was ligated to the 3’ ends of miRNAs, followed by ligation of an RNA adapter to the 5’ end. A reverse transcription primer containing an integrated unique molecular index (UMI) was used to convert the 3′/5′ ligated miRNAs into cDNA. The cDNA was amplified using PCR (22 cycles), and during the PCR indices were added. Afterwards, PCR samples were purified, and library preparation underwent quality control by capillary electrophoresis (Tape D1000, Agilent, Santa Clara, CA, USA). One sample within the AGA subgroup failed the library quality control and was therefore discarded. Based on the quality of the inserts and the concentration measurements, the indexed libraries were pooled in equimolar ratios and sequenced on a NextSeq 2000 machine (Illumina Inc, San Diego, CA, USA) according to the manufacturer instructions, with a 1 × 75 bp read length and an average sequencing depth of 25 million reads per sample. Raw data were demultiplexed, and FASTQ files for each sample were generated with the bcl2fastq2 software v2.20.0.422 (Illumina Inc., San Diego, CA, USA).

### 4.5. Analysis of Sequencing Data

FASTQ files were uploaded to a CLC Genomics Server 23.0.5 (Qiagen) for quality control, alignment and expression quantification. Briefly, the reads were processed by trimming of the common sequence, UMI and adapters and filtering of reads with a length <15 nucleotides or a length >55 nucleotides. The remaining reads were collapsed into UMI counts and aligned sequentially to miRBase v22.1. The UMI counts of each miRNA were quantified and then normalized using the trimmed mean of M-values (TMM) method implemented in the edgeR Bioconductor package [65]. Differential expression of miRNAs was analyzed using DESeq2 (version 1.28.1) with log_2_ fold change ≥1.3 and *p* < 0.05 as the threshold. For each miRNA the log_2_ fold change was calculated from the read count. The results are expressed as the average expression in AGA samples vs. the average expression in SGA samples.

### 4.6. Enrichment Analysis of Predicted miRNA Target Genes

MiRSystem database, a web-based tool that combines DIANA, miRanda, miRBridge, PicTar, PITA, rna22 and TargetScan programs for miRNA target gene prediction, was used to predict potential targets and pathways of the studied miRNAs. The enrichment analysis of gene ontology and associated pathways (KEGG and WIKIPATHWAY) was performed through the DAVID web server.

### 4.7. miRNA Validation by RT-qPCR

The miRNAs more differentially expressed in the AGA versus the SGA subgroups (fold change ≤−2.4 or ≥2.4, n = 8) were validated in the whole cohort (40 AGA; 35 SGA) by means of RT-qPCR. The miRNeasy serum/plasma kit (Qiagen) was used to purify miRNA from 100 µL of isolated exosomes. UniSp2, UniSp4 and UniSp5 RNA from the RNA spike-in kit (Qiagen) were taken as an exogenous control of exosomal miRNAs. Reverse transcription was performed with the miRCURY LNA RT kit (Qiagen) using the exogenous RNA UniSp6 spike-in as a control. miRNA expression was assessed by real-time PCR using miRCURY LNA SYBR Green PCR kit and specific miRCURY LNA miRNA PCR assays (Qiagen). Reactions were run in duplicate on an ABI PRISM 7500 thermal cycler (Applied Biosystems, Waltham, MA, USA) under the following conditions: 2 min polymerase activation at 95 °C, and 40 cycles of 10 s at 95 °C for denaturation and 60 s at 56 °C for annealing and extension. Normalization was performed using the mean expression of miR-23a-3p, miR-101-3p and miR-26a-5p, as they have been reported to be suitable reference genes in extracellular vesicles [66] and showed stable expression in all samples (Table S3). Relative miRNA expression levels were calculated according to the 2^−ΔΔCT^ method.

### 4.8. Statistical Analysis and Ethics

Data were analyzed using GraphPad Software 8.0.2 (La Jolla, CA, USA) and SPSS Statistics 27.0 (IBM Corp., Armonk, NY, USA). Continuous variables are presented as mean ± SEM. To compare the differences between two groups, an unpaired two-tailed Student’s *t*-test was performed for normally distributed variables, and a Mann–Whitney U test was used for non-parametric variables. For binary variables, a Chi-square was used. The associations between cord blood-derived exosomal miRNAs and auxological, metabolic and body composition parameters were assessed using Pearson correlations. Receiver operating characteristic (ROC) curve analysis was conducted to assess the predictive power of differentially expressed miRNAs. The level of significance was set at *p* < 0.05.

The study was approved by the institutional Review Board of Hospital Sant Joan de Déu at the University of Barcelona. Written informed consent was obtained from parents before delivery. All participating families received information about both the nature of the research and how the biological samples would be stored and potentially used in future research projects beyond the present project.

## Figures and Tables

**Table 1:** Longitudinal data (0–12 months) from AGA, (n = 40) and SGA, (n = 35) infants.

	At Birth		4 Months		Δ 0–4 Months		12 Months		Δ 0–12 Months	
	AGA	SGA	AGA	SGA	AGA	SGA	AGA	SGA	AGA	SGA
**Auxology**										
Gestational age (wk)	39.7 ± 0.2	38.5 ± 0.3 ^‡^	--	--	--	--	--	--	--	--
Weight (kg)	3.3 ± 0.1	2.2 ± 0.1 ^‡^	6.6 ± 0.1	6.1 ± 0.1 *	3.3 ± 0.1	3.9 ± 0.1 ^§^	9.8 ± 0.3	9.3 ± 0.3	6.5 ± 0.3	7.1 ± 0.2
Weight Z-score	−0.2 ± 0.1	−2.4 ± 0.1 ^‡^	−0.3 ± 0.2	−1.2 ± 0.2 ^§^	−0.3 ± 0.2	−1.2 ± 0.2 ^§^	−0.1 ± 0.2	−0.5 ± 0.2	0.1 ± 0.2	1.9 ± 0.2 ^‡^
Length (cm)	49.8 ± 0.3	45.8 ± 0.3 ^‡^	62.7 ± 0.4	60.5 ± 0.5 ^§^	12.9 ± 0.5	14.7 ± 0.5 *	74.8 ± 0.7	73.4 ± 0.5	25.0 ± 0.8	27.6 ± 0.5 ^§^
Length SDS	−0.1 ± 0.2	−1.7 ± 0.1 ^‡^	−0.4 ± 0.2	−1.3 ± 0.2 ^§^	−0.3 ± 0.3	0.4 ± 0.2 *	0.0 ± 0.3	−0.5 ± 0.2	0.1 ± 0.4	1.2 ± 0.2 *
BMI (kg/m^2^)	13.2 ± 0.1	10.6 ± 0.1 ^‡^	17.1 ± 0.4	16.6 ± 0.2	3.9 ± 0.3	6.0 ± 0.2 ^‡^	17.5 ± 0.4	17.2 ± 0.3	4.3 ± 0.4	6.6 ± 0.3 ^‡^
BMI Z-score (kg/m^2^)	0.5 ± 0.1	−1.7 ± 0.1 ^‡^	0.2 ± 0.2	−0.2 ± 0.2	−0.3 ± 0.2	1.5 ± 0.2 ^‡^	0.0 ± 0.3	−0.2 ± 0.2	−0.5 ± 0.3	1.5 ± 0.3 ^‡^
Sex (% female)	48	51	--	--	--	--	--	--	--	--
Delivery (% C-section)	16	40 *	--	--	--	--	--	--	--	--
Smoking mothers (%)	17.1	45.4 *	--	--	--	--	--	--	--	--
**Endocrine–metabolic variables**										
Glucose (mmol/L)	4.7 ± 0.2	4.3 ± 0.2	5.0 ± 0.1	4.9 ± 0.1	0.3 ± 0.1	0.6 ± 0.2	4.7 ± 0.1	4.6 ± 0.1	0.0 ± 0.2	0.3 ± 0.2
Insulin (pmol/L)	35 ± 7	28 ± 5	38 ± 6	43 ± 6	3 ± 9	15 ± 9	25 ± 5	29 ± 5	−10 ± 10	1 ± 8
HOMA-IR	1.2 ± 0.3	0.8 ± 0.2	1.3 ± 0.2	1.4 ± 0.2	0.1 ± 0.3	0.6 ± 0.3	0.8 ± 0.2	0.9 ± 0.2	−0.3 ± 0.4	0.1 ± 0.2
IGF-1 (nmol/L)	7.2 ± 0.5	4.7 ± 0.3 ^‡^	5.5 ± 0.4	9.9 ± 1.3 ^‡^	−1.7 ± 0.6	5.2 ± 0.1 ^‡^	7.6 ± 0.7	14.6 ± 1.4 ^‡^	0.4 ± 0.9	9.9 ± 2.0 ^‡^
HMW-adip (mg/L)	32.3 ± 1.8	22.4 ± 2.0 ^‡^	29.8 ± 2.2	38.3 ± 3.2 *	−2.5 ± 2.6	15.9 ± 3.6 ^‡^	17.9 ± 1.5	17.3 ± 1.7	−14.0 ± 2.5	−5.1 ± 2.9 ^§^
**Body Composition (DXA)**										
Fat mass (kg)	0.7 ± 0.1	0.4 ± 0.1 ^‡^	2.6 ± 0.1	2.4 ± 0.1	1.9 ± 0.1	2.0 ± 0.1	3.6 ± 0.1	3.3 ± 0.1	2.9 ± 0.1	2.9 ± 0.1
Abdominal fat (kg)	0.04 ± 0.01	0.02 ± 0.01 ^‡^	0.15 ± 0.01	0.14 ± 0.01	0.11 ± 0.01	0.12 ± 0.01	0.19 ± 0.01	0.18 ± 0.01	0.15 ± 0.16	0.16 ± 0.01
Lean mass (kg)	3.0 ± 0.1	2.3 ± 0.1 ^‡^	4.5 ± 0.1	3.9 ± 0.1 ^§^	1.3 ± 0.1	1.6 ± 0.1	6.7 ± 0.2	6.0 ± 0.2 ^§^	3.7 ± 0.1	3.7 ± 0.2

BMI, body mass index; HOMA-IR, homeostasis model assessment—insulin resistance; IGF-1, insulin-like growth factor 1; HMW-adip, high-molecular-weight adiponectin. Values are mean ± SEM. * *p* < 0.05, ^§^ *p* < 0.01, ^‡^ *p* < 0.001 vs. AGA.

**Table 2:** Pathway enrichment analysis of the predicted target genes of differentially expressed miRNAs.

	Category	Term	Count	*p*-Value
miR-1-3p	KEGG	Focal adhesion	22	3.3 × 10^−5^
	KEGG	PI3k/Akt signaling pathway	30	1.4 × 10^−4^
	KEGG	Neurotrophin signaling pathway	15	2.0 × 10^−4^
	KEGG	Apelin signaling pathway	16	3.0 × 10^−4^
	KEGG	AGE-RAGE signaling pathway in diabetic complications	13	4.7 × 10^−4^
	KEGG	Rap1 signaling pathway	20	5.1 × 10^−4^
	WIKIPATHWAYS	Genes targeted by miRNAs in adipocytes	10	3.2 × 10^−10^
	WIKIPATHWAYS	TGF beta signaling pathway	16	6.0 × 10^−4^
	WIKIPATHWAYS	PI3k/Akt signaling	28	1.7 × 10^−3^
	WIKIPATHWAYS	Pentose phosphate metabolism	4	2.5 × 10^−3^
	WIKIPATHWAYS	Focal adhesion	19	2.6 × 10^−3^
	WIKIPATHWAYS	Insulin signaling	16	4.1 × 10^−3^
miR-133a-3p	KEGG	Adherens junction	8	3.5 × 10^−3^
	KEGG	PI3k/Akt signaling pathway	15	2.1 × 10^−2^
	KEGG	Insulin secretion	6	3.5 × 10^−2^
	KEGG	Endocytosis	11	4.1 × 10^−2^
	KEGG	Neurotrophin signaling pathway	7	4.2 × 10^−2^
	WIKIPATHWAYS	Genes targeted by miRNAs in adipocytes	6	7.6 × 10^−6^
miR-206	KEGG	Rap1 signaling pathway	23	1.2 × 10^−5^
	KEGG	Focal adhesion	22	2.0 × 10^−5^
	KEGG	Apelin signaling pathway	17	6.1 × 10^−5^
	KEGG	Neurotrophin signaling pathway	15	1.4 × 10^−4^
	KEGG	PI3k/Akt signaling pathway	29	1.9 × 10^−4^
	KEGG	AGE-RAGE signaling pathway in diabetic complications	11	4.2 × 10^−3^
	WIKIPATHWAYS	Genes targeted by miRNAs in adipocytes	7	7.9 × 10^−6^
	WIKIPATHWAYS	TGF beta signaling pathway	17	1.6 × 10^−4^
	WIKIPATHWAYS	Focal adhesion	19	2.2 × 10^−3^
	WIKIPATHWAYS	PI3k/Akt signaling	27	2.8 × 10^−3^
	WIKIPATHWAYS	Insulin signaling	16	3.6 × 10^−3^
miR-519d-3p	KEGG	Endocytosis	32	2.7 × 10^−6^
	KEGG	TGF beta signaling pathway	19	5.6 × 10^−6^
	KEGG	MAPK signaling pathway	34	1.5 × 10^−5^
	KEGG	Neurotrophin signaling pathway	18	8.8 × 10^−5^
	KEGG	TNF signaling pathway	17	2.6 × 10^−4^
	KEGG	PI3k/Akt signaling pathway	32	2.2 × 10^−3^
	WIKIPATHWAYS	TGF beta signaling pathway	22	7.0 × 10^−6^
	WIKIPATHWAYS	Insulin signaling	22	1.3 × 10^−4^
miR-372-3p	KEGG	MAPK signaling pathway	29	8.5 × 10^−6^
	KEGG	TGF beta signaling pathway	14	2.0 × 10^−4^
	KEGG	PI3k/Akt signaling	29	2.4 × 10^−4^
	KEGG	Rap1 signaling pathway	19	1.1 × 10^−3^
	KEGG	Axon guidance	17	1.6 × 10^−3^
	KEGG	Endocytosis	20	3.2 × 10^−3^
	KEGG	Focal adhesion	16	9.9 × 10^−3^
	WIKIPATHWAYS	TGF beta signaling pathway	17	1.2 × 10^−4^
	WIKIPATHWAYS	Hippo signaling regulation	12	2.5 × 10^−3^
	WIKIPATHWAYS	Insulin signaling	16	2.8 × 10^−3^
miR-1299 *	KEGG	Hedgehog signaling pathway	13	2.7 × 10^−5^
	KEGG	Insulin resistance	14	4.6 × 10^−3^
	KEGG	AGE-RAGE signaling pathway in diabetic complications	12	1.7 × 10^−2^
	KEGG	Cell adhesion molecules	16	1.9 × 10^−2^
	WIKIPATHWAYS	Hedgehog signaling pathway	13	3.8 × 10^−6^
	WIKIPATHWAYS	Glycosaminoglycan synthesis in fibroblasts	8	8.6 × 10^−3^
	WIKIPATHWAYS	Insulin signaling	17	2.3 × 10^−3^

KEGG pathway enrichment analysis was performed using the predicted targets of the six differentially expressed exosomal miRNAs: miR-1-3p, n = 725; miR-133a-3p, n = 392; miR-206, n = 699; miR-372-3p, n = 814; miR-519d-3p, n = 1000; all targets were predicted by using the miRSystem; miR-1299 *, n = 1119 targets were predicted by miRDB. AGE, advanced glycation end products; KEGG, Kyoto Encyclopedia of Genes and Genomes; RAGE, receptor for advanced glycation end products.

**Table 3:** Bivariate correlations between down-regulated exosomal miRNAs at birth and auxological, endocrine–metabolic and imaging parameters, at birth and throughout follow-up to age 12 mo in AGA (n = 40) and SGA (n = 35).

	At Birth		4 mo		Δ 0–4 mo		12 mo		Δ 0–12 mo	
	r	*p*	R	*p*	r	*p*	r	*p*	R	*p*
**mir-1-3p**										
**Auxology**										
GA	**0.339**	**0.003**	--	--	--	--	--	--	--	--
Weight	**0.596**	**<0.0001**	**0.296**	**0.009**	−0.044	0.706	ns	ns	0.034	0.773
Weight Z-score	**0.638**	**<0.0001**	**0.306**	**0.008**	**−0.406**	**0.0004**	**0.364**	**0.002**	**−0.507**	**<0.0001**
Length	**0.477**	**<0.0001**	**0.313**	**0.006**	−0.116	0.323	0.247	0.034	−0.094	0.425
Length Z-score	**0.490**	**<0.0001**	**0.305**	**0.008**	−0.129	0.268	**0.312**	**0.007**	−0.123	0.296
BMI	**0.544**	**<0.0001**	ns	ns	**−0.346**	**0.002**	ns	ns	**−0.391**	**0.0006**
BMI Z-score	**0.625**	**<0.0001**	ns	ns	**−0.498**	**<0.0001**	ns	ns	**−0.527**	**<0.0001**
**Endocrine–metabolic variables**										
HOMA-IR	0.299	0.019	−0.284	0.019	**−0.344**	**0.008**	**−0.364**	**0.003**	**−0.358**	**0.006**
IGF-1	**0.391**	**0.0006**	**−0.409**	**0.0004**	**−0.460**	**<0.0001**	**−0.410**	**0.0006**	**−0.435**	**0.0002**
HMW-adip	**0.385**	**0.0007**	ns	ns	**−0.361**	**0.008**	0.274	0.031	ns	ns
**DXA**										
Body fat	**0.342**	**0.003**	**0.365**	**0.001**	ns	ns	ns	ns	−0.301	0.011
Abdominal fat	0.273	0.019	ns	ns	ns	ns	ns	ns	−0.262	0.029
Lean mass	**0.548**	**<0.0001**	ns	ns	ns	ns	**0.305**	**0.009**	ns	ns
**mir-133a-3p**										
**Auxology**										
GA	**0.321**	**0.005**	--	--	--	--	--	--	--	--
Weight	**0.501**	**<0.0001**	ns	ns	**−0.259**	**0.028**	ns	ns	**−0.294**	**0.013**
Weight Z-score	**0.479**	**<0.0001**	ns	ns	**−0.377**	**0.001**	ns	ns	**−0.427**	**0.0002**
Length	**0.353**	**0.002**	ns	ns	−0.172	0.139	ns	ns	−0.139	0.238
Length Z-score	**0.338**	**0.003**	ns	ns	−0.131	0.263	ns	ns	−0.129	0.270
BMI	**0.506**	**<0.0001**	ns	ns	**−0.378**	**0.0009**	ns	ns	**−0.384**	**0.0008**
BMI Z-score	**0.495**	**<0.0001**	ns	ns	**−0.402**	**0.0004**	ns	ns	**−0.364**	**0.001**
**Endocrine–metabolic variables**										
HOMA-IR	0.334	0.011	**−0.337**	**0.006**	**−0.343**	**0.007**	**−0.319**	**0.009**	**−0.344**	**0.008**
IGF-1	**0.319**	**0.006**	−0.256	0.029	**−0.345**	**0.003**	**−0.337**	**0.004**	**−0.398**	**0.0008**
HMW-adip	**0.337**	**0.003**	ns	ns	ns	ns	0.294	0.018	ns	ns
**DXA**										
Body fat	**0.338**	**0.003**	ns	ns	ns	ns	ns	ns	−0.258	0.033
Abdominal fat	0.245	0.036	ns	ns	ns	ns	ns	ns	−0.264	0.028
Lean mass	**0.373**	**0.001**	ns	ns	ns	ns	0.251	0.033	ns	ns
**mir-206**										
**Auxology**										
GA	**0.338**	**0.003**	--	--	--	--	--	--	--	--
Weight	**0.594**	**<0.0001**	0.284	0.014	**−0.255**	**0.028**	ns	ns	−0.104	0.378
Weight Z-score	**0.585**	**<0.0001**	**0.321**	**0.005**	**−0.387**	**0.0007**	ns	ns	**−0.399**	**0.0005**
Length	**0.382**	**0.0007**	ns	ns	−0.137	0.239	ns	ns	−0.106	0.371
Length Z-score	**0.369**	**0.001**	0.229	0.048	−0.065	0.577	ns	ns	−0.069	0.555
BMI	**0.625**	**<0.0001**	ns	ns	**−0.433**	**0.0001**	ns	ns	**−0.414**	**0.0003**
BMI Z-score	**0.631**	**<0.0001**	ns	ns	**−0.519**	**<0.0001**	ns	ns	**−0.444**	**<0.0001**
**Endocrine–metabolic variables**										
HOMA-IR	ns	ns	ns	ns	**−0.351**	**0.008**	**−0.328**	**0.008**	**−0.331**	**0.014**
IGF-1	**0.389**	**0.0007**	**−0.308**	**0.009**	**−0.371**	**0.001**	**−0.412**	**0.004**	**−0.491**	**<0.0001**
HMW-adip	**0.335**	**0.004**	ns	ns	ns	ns	ns	ns	ns	ns
**DXA**										
Body fat	**0.459**	**<0.0001**	0.240	0.039	ns	ns	ns	ns	−0.288	0.016
Abdominal fat	**0.387**	**0.0006**	ns	ns	ns	ns	ns	ns	−0.292	0.014
Lean mass	**0.463**	**<0.0001**	ns	ns	ns	ns	**0.353**	**0.003**	ns	ns

GA, gestational age; BMI, body mass index; HOMA-IR, homeostasis model assessment—insulin resistance; IGF-1, insulin-like growth factor-1; HMW-adip, high-molecular-weight adiponectin; ns, not significant. Bivariate correlations with *p* ≤ 0.009 are highlighted in bold.

**Table 4:** Bivariate correlations between up-regulated exosomal miRNAs at birth and auxological, endocrine–metabolic and imaging parameters, at birth and throughout follow-up to age 12 mo in AGA (n = 40) and SGA (n = 35) children.

	At Birth		4 mo		Δ 0–4 mo		12 mo		Δ 0–12 mo	
	r	*p*	R	*p*	r	*p*	r	*p*	R	*p*
**mir-519d-3p**										
**Auxology**										
GA	**−0.305**	**0.009**	--	--	--	--	--	--	--	--
Weight	**−0.425**	**0.0001**	ns	ns	**0.304**	**0.009**	ns	ns	**0.322**	**0.006**
Weight Z-score	**−0.443**	**0.0001**	ns	ns	**0.393**	**0.0006**	ns	ns	**0.447**	**<0.0001**
Length	**−0.303**	**0.008**	ns	ns	ns	ns	ns	ns	0.241	0.042
Length Z-score	**−0.322**	**0.005**	ns	ns	ns	ns	ns	ns	0.239	0.043
BMI	--	--	ns	ns	**0.418**	**0.0003**	ns	ns	**0.394**	**0.0007**
BMI Z-score	**−0.403**	**0.0003**	0.276	0.026	**0.427**	**0.0002**	0.305	0.014	**0.386**	**0.0009**
**Endocrine–metabolic variables**										
HOMA-IR	ns	ns	0.293	0.017	**0.405**	**0.002**	**0.332**	**0.007**	**0.397**	**0.003**
IGF-1	ns	ns	**0.395**	**0.0007**	**0.362**	**0.002**	**0.403**	**0.0008**	**0.389**	**0.001**
HMW-adip	ns	ns	ns	ns	ns	ns	ns	ns	ns	ns
**DXA**										
Body fat	−0.272	0.019	0.250	0.038	**0.320**	**0.006**	ns	ns	**0.313**	**0.009**
Abdominal fat	ns	ns	**0.351**	**0.002**	**0.455**	**<0.0001**	ns	ns	**0.329**	**0.008**
Lean mass	**−0.353**	**0.002**	−0.245	0.036	ns	ns	−0.289	0.016	ns	ns
**mir-372-3p**										
**Auxology**										
GA	−0.241	0.047	--	--	--	--	--	--	--	--
Weight	−0.243	0.037	ns	ns	ns	ns	ns	ns	ns	ns
Weight Z-score	**−0.324**	**0.005**	ns	ns	0.268	0.026	ns	ns	0.283	0.020
Length	ns	ns	ns	ns	0.240	0.042	ns	ns	0.267	0.028
Length Z-score	ns	ns	ns	ns	ns	ns	ns	ns	ns	ns
BMI	**−0.348**	**0.003**	ns	ns	0.264	0.029	ns	ns	0.302	0.012
BMI Z-score	**−0.350**	**0.002**	ns	ns	0.261	0.032	ns	ns	**0.313**	**0.009**
**Endocrine–metabolic variables**										
HOMA-IR	−0.270	0.036	**0.373**	**0.002**	**0.339**	**0.009**	ns	ns	**0.355**	**0.007**
IGF-1	−0.252	0.035	0.256	0.033	**0.326**	**0.007**	0.272	0.025	**0.333**	**0.006**
HMW-adip	ns	ns	ns	ns	ns	ns	ns	ns	ns	ns
**DXA**										
Body fat	ns	ns	**0.309**	**0.008**	**0.323**	**0.006**	ns	ns	0.271	0.027
Abdominal fat	ns	ns	**0.320**	**0.006**	**0.306**	**0.009**	ns	ns	0.257	0.040
Lean mass	ns	ns	ns	ns	ns	ns	ns	ns	ns	ns
**mir-1299**										
**Auxology**										
GA	−0.276	0.022	--	--	--	--	--	--	--	--
Weight	**−0.357**	**0.002**	ns	ns	0.257	0.033	ns	ns	ns	ns
Weight Z-score	**−0.384**	**0.0009**	ns	ns	0.249	0.034	ns	ns	0.299	0.011
Length	−0.283	0.014	ns	ns	0.232	0.046	ns	ns	ns	ns
Length Z-score	**−0.361**	**0.002**	ns	ns	0.276	0.017	ns	ns	ns	ns
BMI	−0.277	0.018	ns	ns	**0.341**	**0.005**	ns	ns	0.270	0.028
BMI Z-score	−0.269	0.021	ns	ns	**0.329**	**0.007**	ns	ns	**0.327**	**0.009**
**Endocrine–metabolic variables**										
HOMA-IR	ns	ns	0.272	0.037	0.3340	0.012	ns	ns	0.296	0.027
IGF-1	ns	ns	ns	ns	**0.396**	**0.0006**	**0.314**	**0.009**	**0.389**	**0.001**
HMW-adip	−0.266	0.025	ns	ns	ns	ns	ns	ns	ns	ns
**DXA**										
Body fat	−0.265	0.027	ns	ns	**0.317**	**0.009**	ns	ns	**0.328**	**0.008**
Abdominal fat	ns	ns	ns	ns	**0.340**	**0.006**	0.301	0.018	0.269	0.029
Lean mass	**−0.384**	**0.0007**	ns	ns	ns	ns	ns	ns	ns	ns

GA, gestational age; BMI, body mass index; HOMA-IR, homeostasis model assessment—insulin resistance; IGF-1, insulin-like growth factor-1; HMW-adip, high-molecular-weight adiponectin; ns, not significant. Bivariate correlations with *p* ≤ 0.009 are highlighted in bold.

## Data Availability

Data have been deposited at Zenodo repository as supporting data values (DOI: 10.5281/zenodo.15497054) and are publicly available as of the date of publication.

## Supplementary Materials

The following supporting information can be downloaded at https://www.mdpi.com/article/10.3390/ijms26146770/s1.
