# Supplementary material for: Cord Blood Exosomal miRNAs from Small-for-Gestational-Age Newborns: Association with Measures of Postnatal Catch-Up Growth and Insulin Resistance
Source: Int J Mol Sci. 2025 Jul 15;26(14):6770. doi: 10.3390/ijms26146770 (PMC12295645; doi:10.3390/ijms26146770)
Supplement: Supplementary file 1 [file ijms-26-06770-s001.zip › ijms-3695220-supplementary.pdf]

Table S1. miRNAs exclusively expressed in cord blood-derived exosomes from appropriate-for-gestational-age (AGA, n=10) infants.

Name	Identifier
hsa-miR-1281	URS000075E7A3
hsa-miR-544b	URS00000EE7F0
hsa-miR-6529-5p	URS0000D544A8
hsa-miR-4472	URS000029B04A
hsa-miR-656-3p	URS0000202460
hsa-miR-377-3p	URS000007F792
hsa-miR-1253	URS000075A7EC
hsa-miR-7977	URS000075A1F7
hsa-miR-1293	URS00001DABC0
hsa-miR-10399-5p	URS0000D4FABD
hsa-miR-6165	URS0000298172
hsa-miR-6777-3p	URS000075E320
hsa-miR-4672	URS000028BB78
hsa-miR-4745-5p	URS00005485E8
hsa-miR-3197	URS000060FA45
hsa-miR-655-3p	URS00005E1A16
hsa-miR-490-3p	URS00001496AE
hsa-miR-410-3p	URS000047E765
hsa-miR-548a-3p	URS000038037E
hsa-miR-371a-5p	URS000025282C
hsa-miR-4737	URS000057DFC6
hsa-miR-6820-3p	URS000075B26F
hsa-miR-3130-5p	URS00005D7C80
hsa-miR-548at-5p	URS000075D269
hsa-miR-381-5p	URS00004A35E2
hsa-miR-518d-3p	URS00001B6361
hsa-miR-4632-5p	URS000075A071
hsa-miR-4708-5p	URS0000122F23
hsa-miR-4731-5p	URS00005E08E8
hsa-miR-10526-3p	URS0000D52683
hsa-miR-6505-5p	URS000075D8DA
hsa-miR-3186-5p	URS000075C3AF
hsa-miR-6732-3p	URS000075DD0B
hsa-miR-6727-3p	URS000075DDC6
hsa-miR-548h-5p	URS00003E16D3
hsa-miR-3679-3p	URS00003E3F4E
hsa-miR-559	URS000075AE29
hsa-miR-7856-5p	URS000075BCE8
hsa-miR-4662a-5p	URS00005140D0
hsa-miR-2114-3p	URS000075BFBB
hsa-miR-4426	URS00002731AC

hsa-miR-4651	URS00005F9738
hsa-miR-133b	URS000032BD73
hsa-miR-12135	URS0000D52303
hsa-miR-450b-3p	URS00002FF522
hsa-miR-3200-3p	URS0000381B86
hsa-miR-3158-3p	URS000040D577
hsa-miR-3666	URS000075B762
hsa-miR-215-3p	URS000075D75C
hsa-miR-4649-5p	URS000044FB51
hsa-miR-1197	URS00003E5E03
hsa-miR-1973	URS00005030C1
hsa-miR-2277-5p	URS00000D6C3F
hsa-miR-1538	URS00005235AA
hsa-miR-1468-5p	URS00002ECEE4
hsa-miR-577	URS00004CD810
hsa-miR-5100	URS0000079F78
hsa-miR-651-3p	URS000075D3EF
hsa-miR-642b-3p	URS0000453664
hsa-miR-4706	URS00000C403E
hsa-miR-3622b-5p	URS000075E791
hsa-miR-1267	URS000075AEB2
hsa-miR-642a-5p	URS00000F2C33
hsa-miR-212-3p	URS00001D6BAE
hsa-miR-1237-3p	URS000075A763
hsa-miR-4742-3p	URS00000C48FA
hsa-miR-6737-5p	URS000075C8FF
hsa-miR-6796-5p	URS0000759E61
hsa-miR-4653-3p	URS000035646E
hsa-miR-6797-3p	URS000075AB1D
hsa-miR-6790-5p	URS000075A707
hsa-miR-106a-3p	URS00005F5B9E
hsa-miR-1227-3p	URS000075CFA8
hsa-miR-544a	URS00001934CD
hsa-miR-6888-5p	URS000075DC2D
hsa-miR-520a-3p	URS0000101689
hsa-miR-561-3p	URS000075D1DD
hsa-miR-6726-3p	URS000075C89D
hsa-miR-3654	URS000075C695
hsa-miR-4442	URS00003AD80B
hsa-miR-376a-2-5p	URS00004B1322
hsa-miR-24-2-5p	URS00001DEE11
hsa-miR-4781-3p	URS0000128A0A
hsa-miR-378j	URS000075DCB5
hsa-miR-103a-2-5p	URS000060A0B0
hsa-miR-3115	URS00000BB229
hsa-miR-5708	URS000075CEDD

hsa-miR-510-3p	URS000075B179
hsa-miR-6768-5p	URS000075B489
hsa-miR-6510-5p	URS000075A608
hsa-miR-4476	URS000075B0F6
hsa-miR-874-5p	URS000073891E
hsa-miR-6792-5p	URS000075D9C4
hsa-miR-3620-3p	URS000075C163
hsa-miR-4742-5p	URS0000473E7A
hsa-miR-3911	URS00001B968D
hsa-miR-3941	URS000075CA3B
hsa-miR-513b-5p	URS0000284586
hsa-miR-3685	URS0000759D24
hsa-miR-6870-5p	URS000075D04E
hsa-miR-7106-5p	URS000075AD04
hsa-miR-5591-5p	URS000075C971
hsa-miR-3126-5p	URS0000022AAA
hsa-miR-4774-5p	URS000055153D
hsa-miR-4793-3p	URS00001A1CA6
hsa-miR-7110-3p	URS000075CF8A
hsa-miR-6889-5p	URS000075C76B
hsa-miR-4802-5p	URS000044D459
hsa-miR-4758-5p	URS0000378EED
hsa-miR-887-5p	URS000075B4E7
hsa-miR-3161	URS00000D5659
hsa-miR-4444	URS00002A82B3
hsa-miR-10401-5p	URS0000D51CA4
hsa-miR-6726-5p	URS000075C413
hsa-miR-3150a-5p	URS00004D5A7A
hsa-miR-548m	URS000075ECFB
hsa-miR-1261	URS00003975B5
hsa-miR-215-5p	URS0000315B13
hsa-miR-3145-3p	URS00004060DD
hsa-miR-323b-5p	URS000075D04C
hsa-miR-4726-5p	URS0000026924
hsa-miR-5188	URS00004663C8
hsa-miR-548j-3p	URS0000759BFD
hsa-miR-6856-5p	URS000075A8A4
hsa-miR-4656	URS00003F7B61

Table S2. miRNAs exclusively expressed in cord blood-derived exosomes from small-for-gestational-age (SGA, n=10) infants.

Name	Identifier
hsa-let-7e-3p	URS0000409B45
hsa-miR-519e-5p	URS000075AC86
hsa-miR-5697	URS000075CD80
hsa-miR-526b-3p	URS000038B25B
hsa-miR-6890-5p	URS000075AA8B
hsa-miR-378a-5p	URS00004BAA8A
hsa-miR-130b-5p	URS000032A4F7
hsa-miR-4754	URS000058C0AB
hsa-miR-6731-5p	URS000075BFCB
hsa-miR-4786-5p	URS000023BDD3
hsa-miR-3117-3p	URS0000507F3E
hsa-miR-340-3p	URS000048521E
hsa-miR-6126	URS000075D118
hsa-miR-3678-3p	URS000075BC79
hsa-miR-4743-5p	URS0000117FAF
hsa-miR-889-5p	URS000075AAF0
hsa-miR-6868-3p	URS000075DF84
hsa-miR-548ay-3p	URS000075D0C5
hsa-miR-6759-5p	URS000075A5C0
hsa-let-7f-2-3p	URS00001C04A2
hsa-miR-219a-5p	URS0000565C8D
hsa-miR-1469	URS0000539433
hsa-miR-7109-5p	URS000075EDFB
hsa-miR-548aq-3p	URS0000118224
hsa-miR-4680-3p	URS00005CFD69
hsa-miR-6778-5p	URS000075B2C3
hsa-miR-548o-3p	URS0000080D0A
hsa-miR-491-5p	URS00001919B0
hsa-miR-4504	URS000075AB46
hsa-miR-519e-3p	URS00004F4C18
hsa-miR-3690	URS0000330DDA
hsa-miR-6853-3p	URS000075D50A
hsa-miR-708-5p	URS000019D79B
hsa-miR-193b-3p	URS00000AA464
hsa-miR-6125	URS000075F0F0
hsa-miR-1277-3p	URS000044878A
hsa-miR-512-5p	URS0000062B37
hsa-miR-135a-2-3p	URS000075AB6F
hsa-miR-3163	URS00003A5E54
hsa-miR-589-3p	URS00005F9DAE

hsa-miR-1306-3p	URS000041E780
hsa-miR-365b-5p	URS00003E2232
hsa-miR-3122	URS000032AE90
hsa-miR-3173-5p	URS00004216E6
hsa-miR-6840-3p	URS000075C594
hsa-miR-217-5p	URS000041E210
hsa-miR-7114-5p	URS000075E8C6
hsa-miR-7852-3p	URS000075B643
hsa-miR-4659b-3p	URS0000327F71
hsa-miR-490-5p	URS00004556E5
hsa-miR-6746-5p	URS000075AF8F
hsa-miR-3648	URS0000454FAB
hsa-miR-521	URS00001DBB42
hsa-miR-548ap-3p	URS0000578557
hsa-miR-503-3p	URS00003C11EC
hsa-miR-4674	URS000038E667
hsa-miR-1912-3p	URS00004A9320
hsa-miR-523-3p	URS00001383CF
hsa-miR-3074-3p	URS000046F124
hsa-miR-301b-3p	URS0000251D0B
hsa-miR-4781-5p	URS00000913AC
hsa-miR-1295b-3p	URS000075AF37
hsa-miR-11401	URS0000D56AF1
hsa-miR-3201	URS000075A82C
hsa-miR-6753-5p	URS000075EF1E
hsa-miR-511-5p	URS000028DE3F
hsa-miR-6757-5p	URS0000759C08
hsa-miR-676-5p	URS000075F0C5
hsa-miR-1827	URS000056215C
hsa-miR-214-5p	URS00004DAA89
hsa-miR-5687	URS000075E517
hsa-miR-3925-5p	URS0000249054
hsa-miR-3155a	URS00002D349B
hsa-miR-6820-5p	URS000075E8D5
hsa-miR-5006-5p	URS000075CF59
hsa-miR-4520-3p	URS00002A0C10
hsa-miR-4270	URS00005E80AD
hsa-miR-4746-5p	URS0000156390
hsa-miR-4520-2-3p	URS0000486E88
hsa-miR-4503	URS000075A348
hsa-miR-6733-5p	URS000075EA52
hsa-miR-627-3p	URS000075D437
hsa-miR-4797-3p	URS00004848FF

hsa-miR-4729	URS000024F41A
hsa-miR-584-3p	URS000006BE7D
hsa-miR-3065-3p	URS000029D793
hsa-miR-935	URS000033EBB8
hsa-miR-4799-5p	URS00001B09C2
hsa-miR-19b-1-5p	URS00001B9622
hsa-miR-95-5p	URS000075D912
hsa-miR-380-5p	URS000075BE5F
hsa-miR-6798-3p	URS000075C554
hsa-miR-4512	URS0000247AC6
hsa-miR-1249-5p	URS0000782078
hsa-miR-4789-3p	URS00002D6EA8
hsa-miR-4531	URS0000034450
hsa-miR-6770-3p	URS000075D6C4
hsa-miR-24-1-5p	URS00002D0FC3
hsa-miR-7844-5p	URS000075B7DF
hsa-miR-4659a-3p	URS00003003B4
hsa-miR-1193	URS000002F2FA
hsa-miR-6887-5p	URS000075BEF5
hsa-miR-378e	URS00000B1B47
hsa-miR-6851-5p	URS000075ACF5
hsa-miR-6772-5p	URS000075990B
hsa-miR-4534	URS000075B60F
hsa-miR-4705	URS00004FE139
hsa-miR-6875-5p	URS000075DAC6
hsa-miR-4671-3p	URS00005DEA88
hsa-miR-6834-5p	URS000075DF58
hsa-miR-539-3p	URS000039607D
hsa-miR-4795-5p	URS000045C62E
hsa-miR-548u	URS0000358C16
hsa-miR-4259	URS00007E453F
hsa-miR-4762-3p	URS0000437512
hsa-miR-4690-5p	URS00005B6CAF
hsa-miR-4447	URS000075E94B
hsa-miR-34b-5p	URS0000432971
hsa-miR-6130	URS000075DDBE
hsa-miR-1909-3p	URS000009A9A2
hsa-miR-551b-3p	URS000008C563
hsa-miR-4685-3p	URS00001A5096
hsa-miR-500b-5p	URS00000CC005
hsa-miR-6835-5p	URS000075A5BD
hsa-miR-4507	URS0000099F48
hsa-miR-4658	URS00002947E6

hsa-miR-548ar-3p	URS00004283FE
hsa-miR-6732-5p	URS000075DB80
hsa-miR-6787-3p	URS000075CC55
hsa-miR-4700-5p	URS0000334286
hsa-miR-4664-5p	URS00005A06DD
hsa-miR-487a-3p	URS000016FD1B
hsa-miR-132-5p	URS0000028BB8
hsa-miR-11181-3p	URS0000754CC5
hsa-miR-208a-3p	URS00000E5433
hsa-miR-4701-3p	URS000006983A
hsa-miR-33b-3p	URS00001270D3
hsa-miR-4733-3p	URS0000482D79
hsa-miR-1914-5p	URS000075C11C
hsa-miR-181b-3p	URS0000229622
hsa-let-7c-3p	URS000060A34C
hsa-miR-186-3p	URS000021D7C8
hsa-miR-937-5p	URS0000776393
hsa-miR-487a-5p	URS000075BB46
hsa-miR-4688	URS000032D69B
hsa-miR-586	URS000075D29B
hsa-miR-135a-5p	URS00000DF6D0
hsa-miR-1234-3p	URS000075E194
hsa-miR-1286	URS00002EF20A
hsa-miR-4697-3p	URS00003685CF
hsa-miR-1287-3p	URS0000759A79
hsa-miR-2116-5p	URS00000BA274
hsa-miR-3121-3p	URS000052EA98
hsa-miR-329-5p	URS000075BD0A
hsa-miR-3940-5p	URS00001E8DA7
hsa-miR-4457	URS000075AB35
hsa-miR-451b	URS000022EC48
hsa-miR-4526	URS0000009265
hsa-miR-5582-3p	URS000075D126
hsa-miR-6512-5p	URS000075DDC9
hsa-miR-3187-5p	URS0000486E0B
hsa-miR-4768-3p	URS00002B3F70
hsa-miR-6729-3p	URS000075D5F0
hsa-miR-4800-3p	URS0000149319
hsa-miR-550b-2-5p	URS000009C1E9
hsa-miR-5088-3p	URS000075C587
hsa-miR-4505	URS000075EBEE
hsa-miR-1256	URS0000098B3B
hsa-miR-449b-5p	URS00003758F0

hsa-miR-6780b-3p	URS000075C2D2
hsa-miR-617	URS000075B33B
hsa-miR-4645-3p	URS0000212118
hsa-miR-1284	URS00004959F4
hsa-miR-3667-3p	URS000075E2C3
hsa-miR-1247-5p	URS000057DF36
hsa-miR-3691-3p	URS000053E475
hsa-miR-6731-3p	URS000075C778
hsa-miR-3937	URS000075BC71
hsa-miR-7705	URS000060B2B9
hsa-miR-4670-3p	URS00005CC60E
hsa-miR-148b-5p	URS00005A7A84
hsa-miR-362-3p	URS00003A19A3
hsa-miR-548bb-5p	URS00007E3B9B
hsa-miR-549a-3p	URS00004C689A
hsa-miR-6134	URS000075DB54
hsa-miR-4772-5p	URS0000411752
hsa-miR-7111-5p	URS000075EE2D
hsa-miR-6848-5p	URS000075E309
hsa-miR-933	URS0000425000
hsa-miR-5581-3p	URS00001E2F7A
hsa-miR-514b-5p	URS00003AA851
hsa-miR-5706	URS0000381CEC
hsa-miR-6894-3p	URS000075D303
hsa-miR-6842-3p	URS000075B6FC
hsa-miR-6728-5p	URS000075A56F

Figure S1A. Receiver operating characteristic (ROC) curves depicting the discriminatory effect of miRNAs to differentiate appropriate-for-gestational-age (AGA, n=40) and small-for-GA (SGA, n=35) infants.

Down-regulated

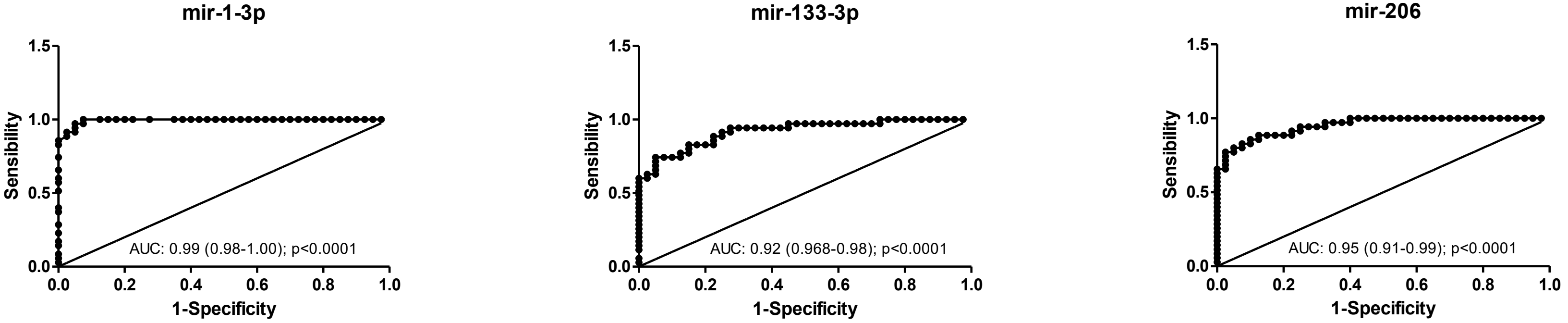

Up-regulated

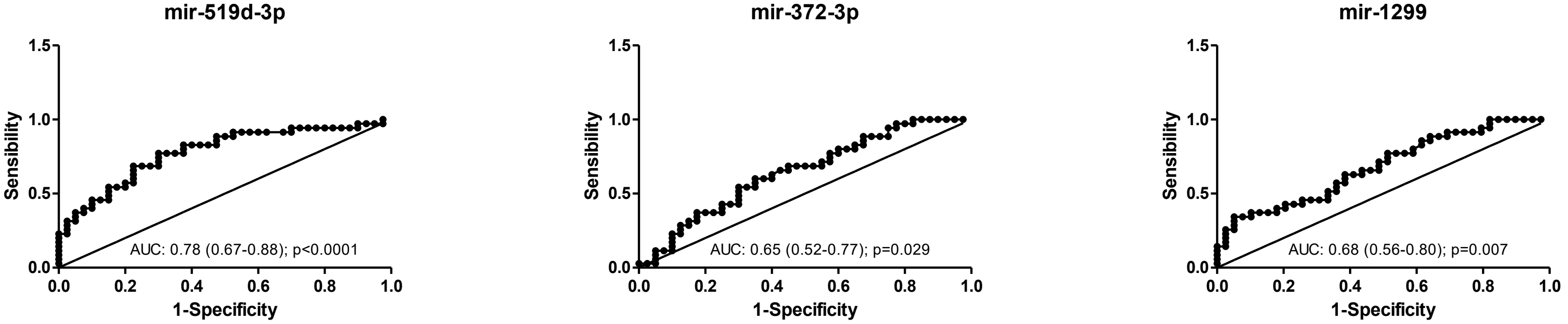

Figure S1B. ROC curves depicting the discriminatory capacity of differentially expressed exosomal miRNAs for predicting the changes in body mass index between birth and age 1 year in AGA (n=40) and SGA (n=35) infants.

Down-regulated

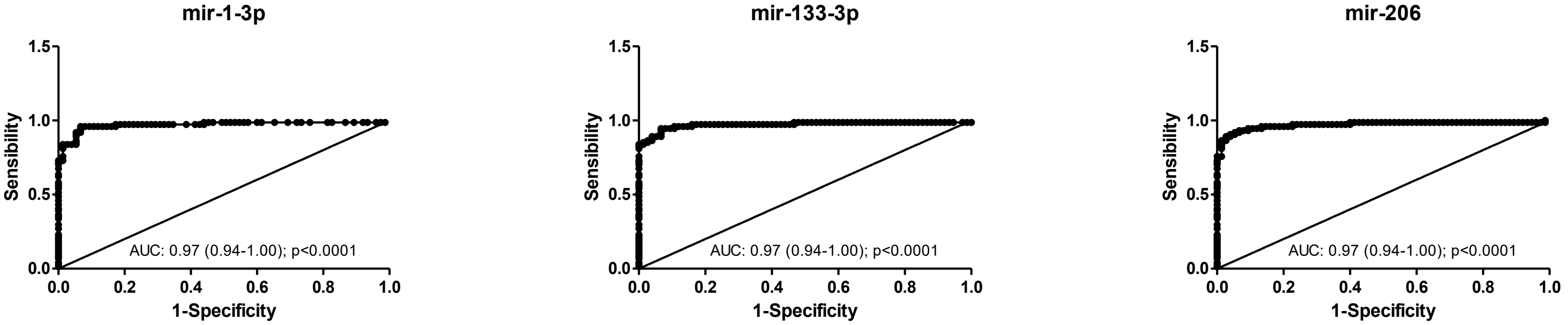

Up-regulated

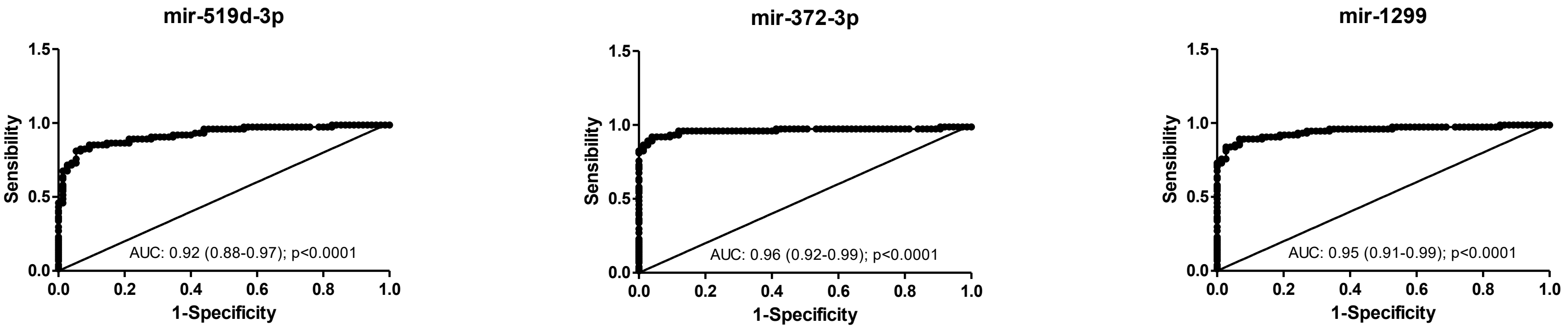

Figure S1C. ROC curves depicting the discriminatory capacity of differentially expressed exosomal miRNAs for predicting the changes in fat mass between birth and age 1 year in AGA (n=40) and SGA (n=35) infants.

Down-regulated

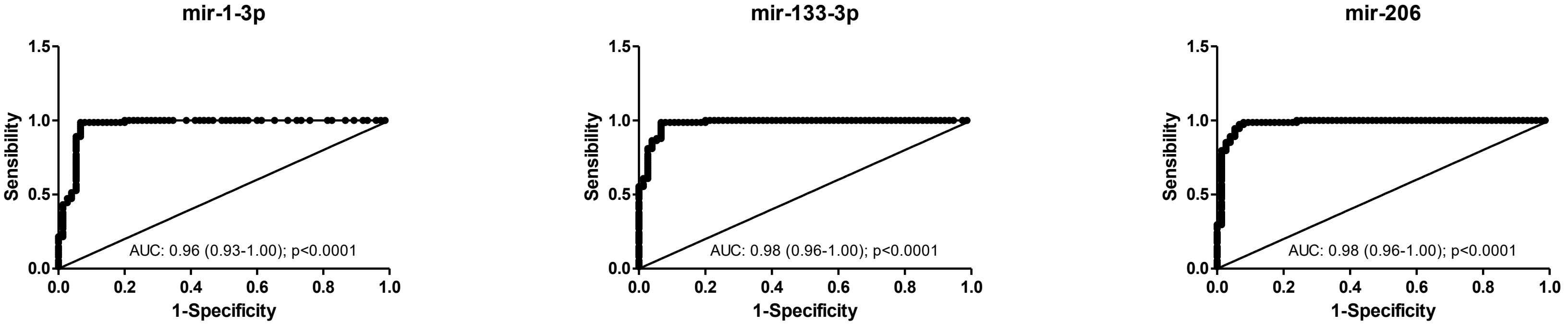

Up-regulated

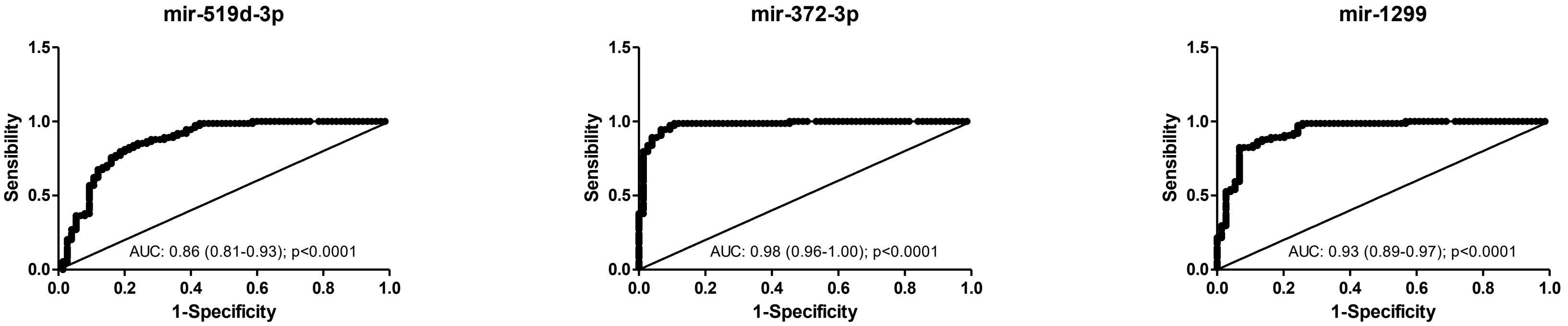

Figure S1D. ROC curves depicting the discriminatory capacity of differentially expressed exosomal miRNAs for predicting the changes in abdominal fat between birth and age 1 year in AGA (n=40) and SGA (n=35) infants.

Down-regulated

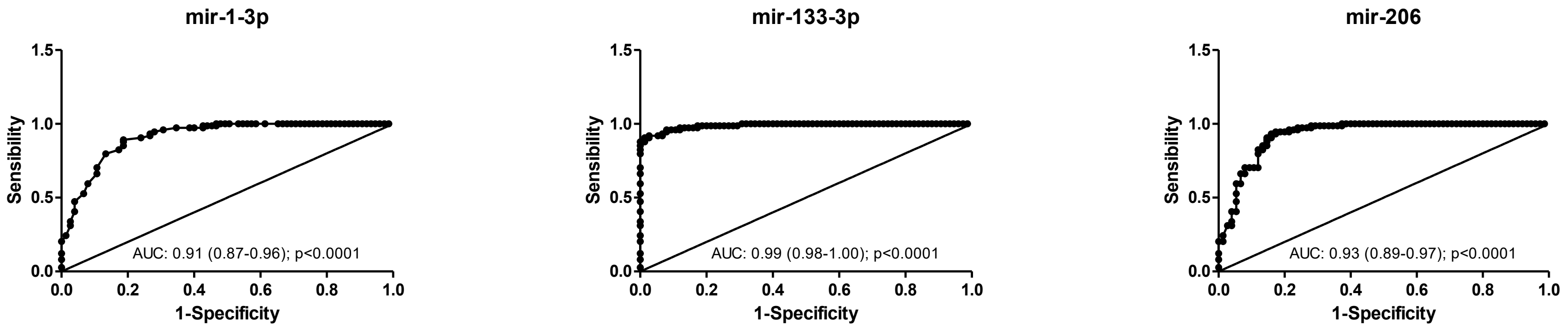

Up-regulated

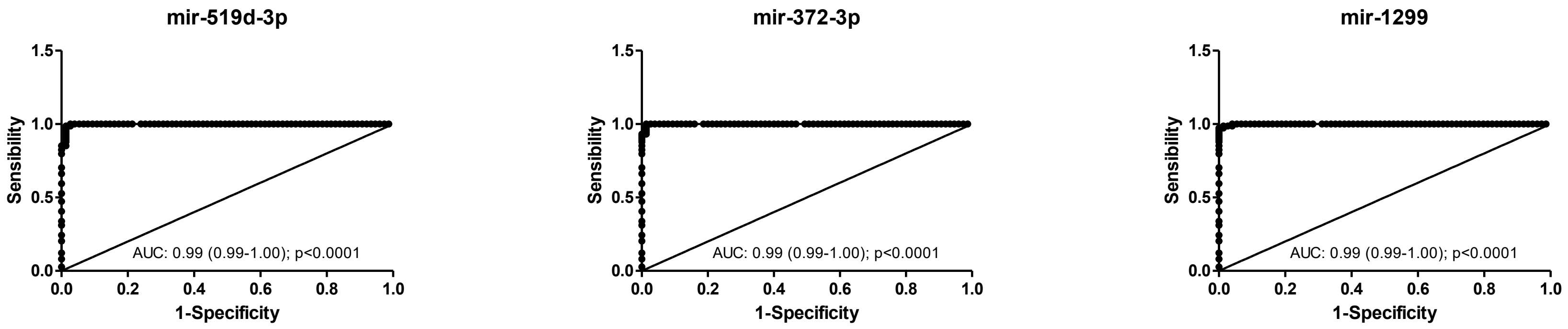

Figure S1E. ROC curves depicting the discriminatory capacity of differentially expressed exosomal miRNAs for predicting the changes in lean mass between birth and age 1 year in AGA (n=40) and SGA (n=35) infants.

Down-regulated

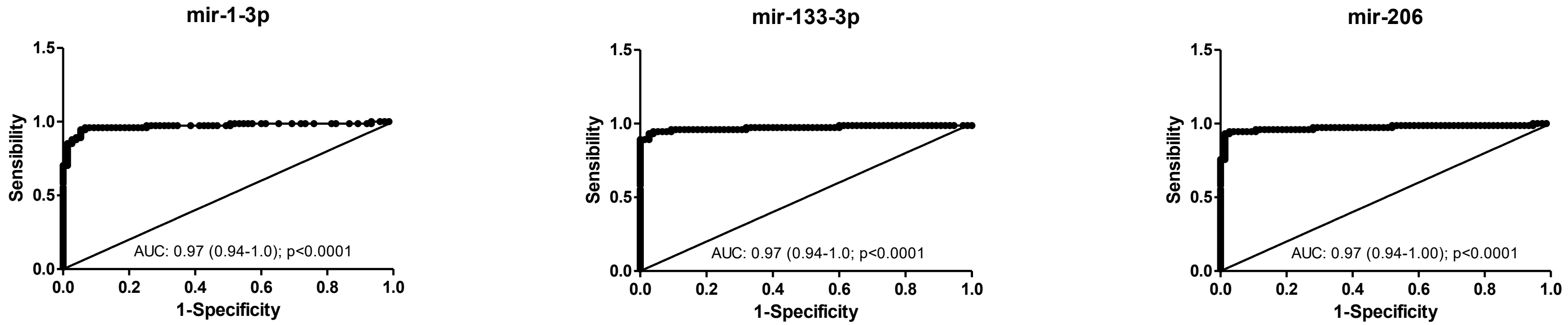

Up-regulated

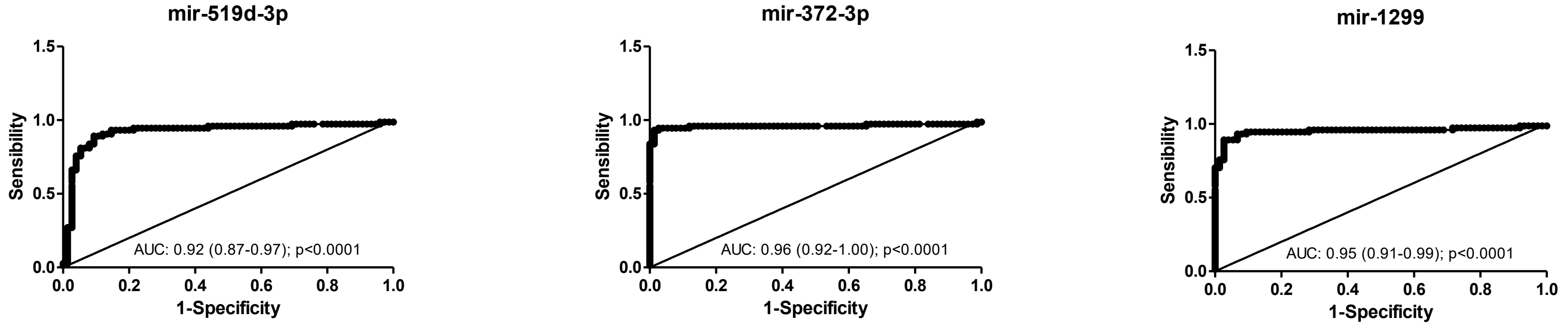

Figure S1F. ROC curves depicting the discriminatory capacity of differentially expressed exosomal miRNAs for predicting the changes in HOMA-IR between birth and age 1 year in AGA (n=40) and SGA (n=35) infants.

Down-regulated

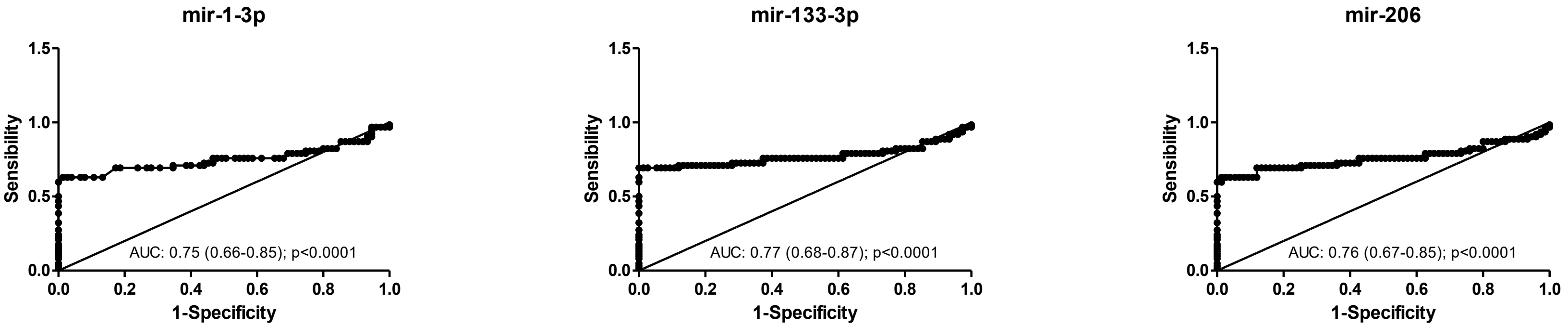

Up-regulated

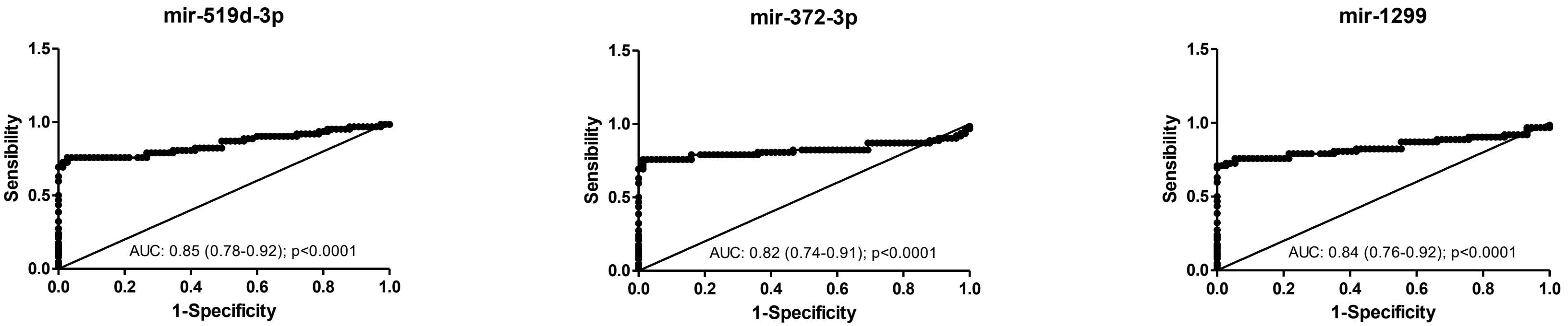

Figure S2. Consecutive recruitment of the appropriate-for-gestational-age (AGA) and small-for-GA (SGA) subpopulations.

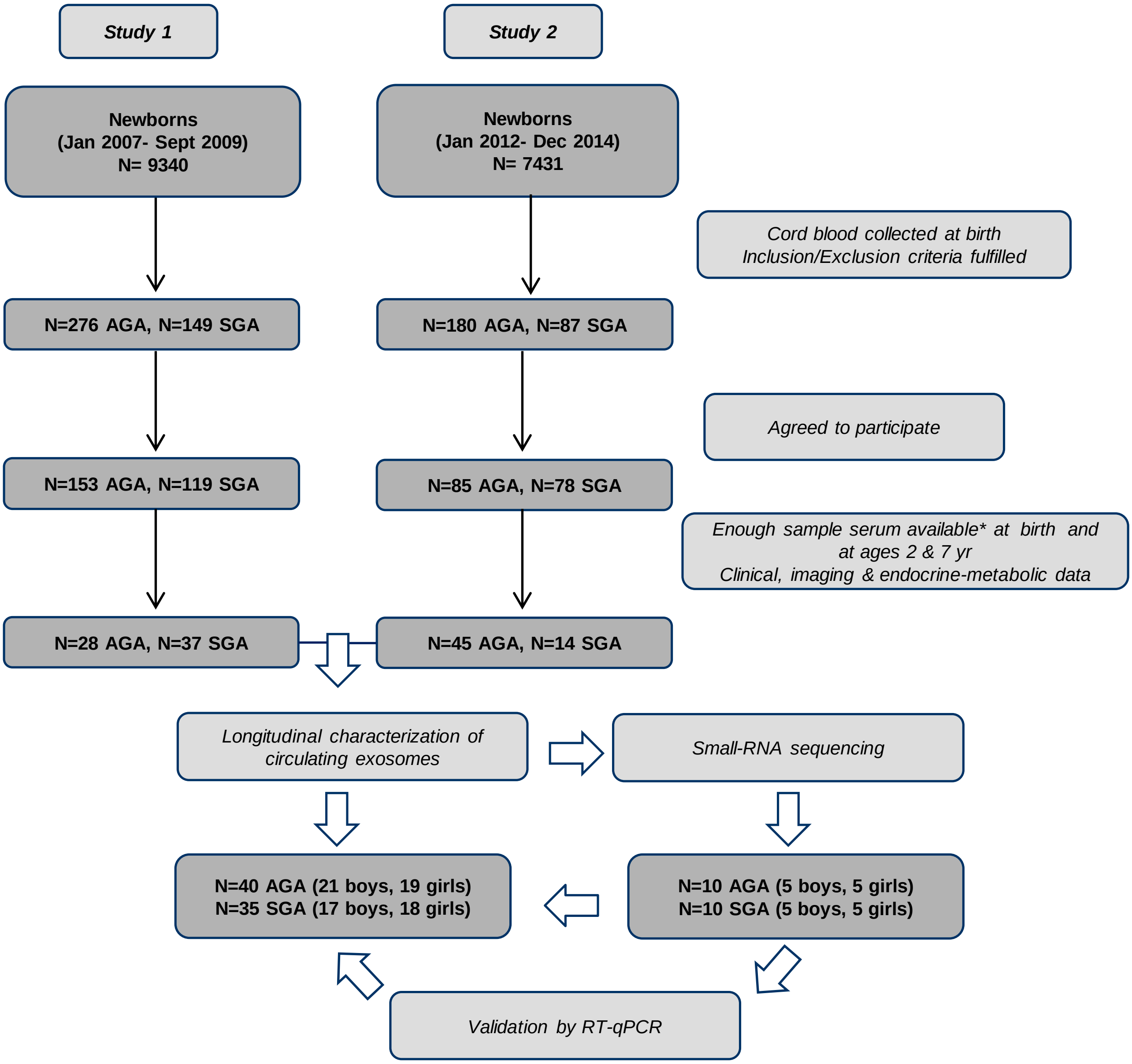

AGA: Appropiate-for-gestational-age
SGA: Small-for-gestational-age
*A minimum of 250 µl of serum is needed for exosome purification

Table S3. Expression levels of the reference genes (mean Ct values) used for RT-qPCR data normalization in cord blood-derived exosomes of infants born appropiate-for-gestational-age [AGA (n=40)] or small-for-gestational-age [SGA (n=35)].

	**AGA** mean Ct			**SGA** mean Ct		
	**miR-23a-3p**	**miR-101-3p**	**miR-26a-5p**	**miR-23a-3p**	**miR-101-3p**	**miR-26a-5p**
**sample**						
1	27.27	28.18	27.48	27.25	29.10	27.03
2	27.37	28.07	27.21	26.97	28.95	25.84
3	26.71	28.21	27.67	26.96	28.49	25.91
4	27.25	28.20	26.10	27.10	28.31	26.53
5	27.31	28.29	26.64	27.15	28.47	26.65
6	27.42	28.47	26.86	27.31	29.50	26.56
7	26.68	28.24	27.13	28.10	29.63	26.80
8	26.89	28.23	26.81	27.27	30.16	25.98
9	26.98	29.13	27.01	27.23	28.17	27.10
10	27.21	24.14	26.10	26.95	29.00	27.14
11	27.33	28.24	26.51	28.21	28.80	27.03
12	27.20	28.28	26.38	26.80	28.85	26.54
13	27.29	28.12	26.46	28.11	28.69	26.67
14	28.21	28.09	26.06	27.20	28.54	25.69
15	26.97	28.24	26.71	26.99	28.82	25.79
16	27.15	29.07	26.90	26.86	29.43	26.44
17	27.09	29.16	26.46	27.21	29.27	26.52
18	27.21	28.10	26.35	28.07	29.16	26.59
19	27.30	28.39	25.87	28.12	28.54	25.71
20	27.13	28.27	25.64	26.91	29.02	25.96
21	26.88	28.41	26.38	27.14	28.20	27.04
22	26.81	29.06	26.44	26.91	28.27	25.67
23	27.22	28.92	26.11	27.97	29.50	26.93
24	27.11	28.86	25.60	28.05	28.66	25.76
25	27.22	28.70	26.46	27.22	28.80	25.85
26	27.27	28.42	27.04	28.11	29.27	27.04
27	26.90	28.30	26.98	28.20	28.39	25.78
28	27.12	28.47	25.86	27.88	30.37	26.89
29	26.26	28.31	25.92	26.79	28.45	25.40
30	26.41	28.02	27.05	26.97	28.59	26.75
31	27.05	28.82	26.12	26.99	28.40	26.88
32	26.85	28.28	26.82	26.91	29.19	27.02
33	27.01	28.17	26.74	28.04	30.27	26.99
34	27.20	28.31	25.84	28.13	29.14	26.84
35	27.07	29.21	26.12	28.21	28.35	26.96
36	27.24	29.27	25.84			
37	28.07	28.14	26.50			
38	26.95	28.78	27.43			
39	27.10	28.60	25.89			
40	27.16	28.50	26.81			
**Mean**	**27.12**	**28.36**	**26.50**	**27.43**	**28.93**	**26.46**
**sem**	**0.05**	**0.12**	**0.08**	**0.09**	**0.10**	**0.09**
